# High Performance Zn–Mn Cement Batteries for the Next Generation of Buildings

**DOI:** 10.1007/s40820-026-02122-x

**Published:** 2026-03-13

**Authors:** Zhaolong Liu, Pan Feng, Long Yuan, Ruidan Liu, Xiangyu Meng, Guanghui Tao, Jian Chen, Zaiping Guo, Changwen Miao

**Affiliations:** 1 State Key Laboratory of Engineering Materials for Major Infrastructure, Southeast University, Nanjing, 210008 People’s Republic of China; 2 School of Chemical Engineering and Advanced Materials, The University of Adelaid, Adelaide, SA 5005 Australia

**Keywords:** Structural energy storage, Active cementitious separator, Zn–Mn batteries, Energy storage mechanism, Capacity enhancement

## Abstract

Integrating energy storage into buildings through cement-based structural batteries offers a transformative pathway toward net-zero energy infrastructure. However, current cementitious structural batteries remain hampered by low energy density and poor cycle stability, largely due to the presumed electrochemical inertness of cement and severe side reactions in the alkaline cementitious environment. Herein, we identify the unexplored role of cement as functional separators containing ZnSO_4_ + MnSO_4_ (Zn–Mn) electrolyte, which facilitates the MnO_2_ deposition on cathode during charging and enhances capacity. Continuously generated zinc sulfate hydroxide within the cement matrix acts as a proton buffer, consuming H^+^ generated during the electrochemical oxidation of Mn^2+^. This buffering prevents local acidification and sustains birnessite-MnO₂ deposition, typically hindered in conventional neutral Zn–Mn electrolytes. This discovery leads to the concept of active cementitious separators for fabricating Zn–Mn cement batteries that combine improved compressive strength (~ 20 MPa) with high specific energy density (0.92 mWh cm^−2^ at 1.15 mW cm^−2^) and excellent cycling stability (99.98% capacity retention after 1000 cycles). Our findings overturn the long-standing perception of cementitious materials as merely passive electrolyte carriers, demonstrating a ten-fold increase in both energy density and cycling stability over previous cement-based batteries.

**Supplementary Information:**

The online version contains supplementary material available at 10.1007/s40820-026-02122-x.

## Highlights

- Conventional cementitious materials were engineered into active cementitious separators (ACSs) that function as capacity boosters rather than passive carriers, significantly enhancing the capacity of Zn–Mn batteries.
- Continously generated zinc sulfate hydroxide within ACSs acts as an effective proton buffer, suppressing electrolyte acidification and stabilizing birnessite-MnO_2_ deposition.
- ACSs-based Zn–Mn batteries achieve a balanced integration of structural integrity and electrochemical performance, delivering a ten-fold improvement in energy density (0.92 mWh cm^−2^ at 1.15 mW cm^−2^) and exceptional cycling stability (99.98% capacity retention after 1000 cycles).

**Supplementary Information:**

The online version contains supplementary material available at 10.1007/s40820-026-02122-x.

## Graphical abstract

**Figure:**
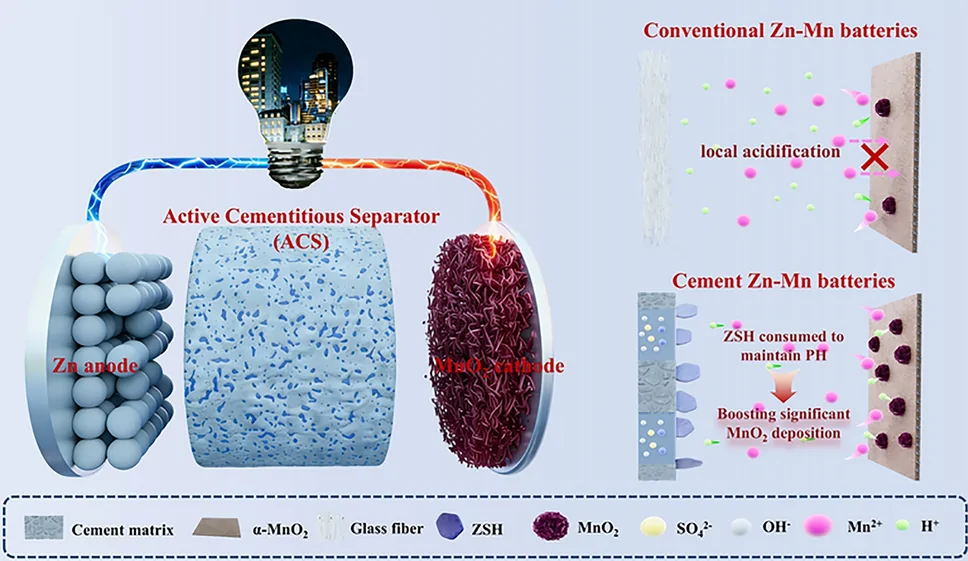

## Introduction

The buildings and construction sector are by far the largest source of global greenhouse gas emissions, accounting for a staggering 37% of the total [1, 2]. As a proactive carbon reduction technique, integrating renewable energy into buildings presents a powerful pathway toward deep carbon reduction and zero-emission infrastructure [3, 4]. However, renewable sources like photovoltaics suffer from low utilization efficiency due to the mismatch between peak power generation and electricity demand. This highlights the urgent need for effective energy storage solutions in buildings [5, 6]. Unlike standalone lithium battery storage systems, structural energy storage systems (SESS) based on widely used cementitious materials offer a low-cost, space-efficient and inherently safe alternative, paving the way for the next generation of energy-storing buildings [7–9].

In SESSs, cementitious materials typically function as porous separators, positioned between electrodes to facilitate ion transport [10, 11]. Most existing electrodes, which rely on electric double-layer capacitance and pseudocapacitance, are commonly used in supercapacitors [12–14]. While these systems offer excellent cycle stability, they generally exhibit low energy density, lack a discharge voltage plateau and come with high costs. In contrast, battery-based SESSs, which store energy through Faradic reactions, provide significantly higher energy density, stable voltage output and reduced cost—key attributes for scalable energy storage in buildings and infrastructure [15].

Recent efforts have explored battery-based SESSs by pairing faradic electrodes such as Fe, Zn, Ni, Cu, Mn and their oxides with cementitious materials to fabricate full batteries [16–18]. However, the inherently alkaline environment and low ionic conductivity of cement severely limit the electrochemical performance of these electrodes [19–21]. More critically, the intrinsic electrochemical activity of cement itself has long been overlooked in material design. As a result, conventional designs suffer from inefficient utilization of active materials and rapid capacity degradation. For instance, the best-performing battery-based SESS reported to date—incorporating a nickel oxide-loaded carbon fiber mesh cathode (250 mg cm^−2^)—achieved an extremely low energy density (3.0 Wh kg^−1^, based on active materials mass) and poor cycling stability (63.4% capacity retention after 100 cycles) [18]. Addressing these challenges requires a fundamental rethinking of cement’s role in electrochemical reactions—specifically, unlocking its intrinsic activity to enable direct participation in redox processes. By harnessing this overlooked potential, cement can contribute to electrochemical reactions, significantly improving active material utilization and enhancing overall energy storage performance.

Given the presence of water-based pore solution in cementitious materials, aqueous batteries have emerged as a promising candidate for SESSs. Among them, zinc anodes offer high theoretical capacities (820 mAh g^−1^ and 5855 Ah L^−1^), low electrochemical potentials (− 0.76 V vs. standard hydrogen electrode) and abundant availability [22–24], making them particularly attractive for large-scale electrochemical energy storage applications. Recent advances have demonstrated the successful integration of aqueous zinc-ion batteries into SESSs by employing cementitious separators infused with ZnSO_4_ electrolyte, striking a balance between ionic conductivity and mechanical strength [8]. Moreover, hydrated cementitious materials contain alkaline oxides and hydrates that readily react with acidic species. This intrinsic property aligns with the recently recognized pH-buffering effect, which promotes formation of active Mn-based deposits by eliminating H^+^ generated in the oxidative process of Mn^2+^ [25–28]. Such synergy opens a promising pathway toward high-performance Zn–Mn battery-based SESSs.

**Fig. 1:**
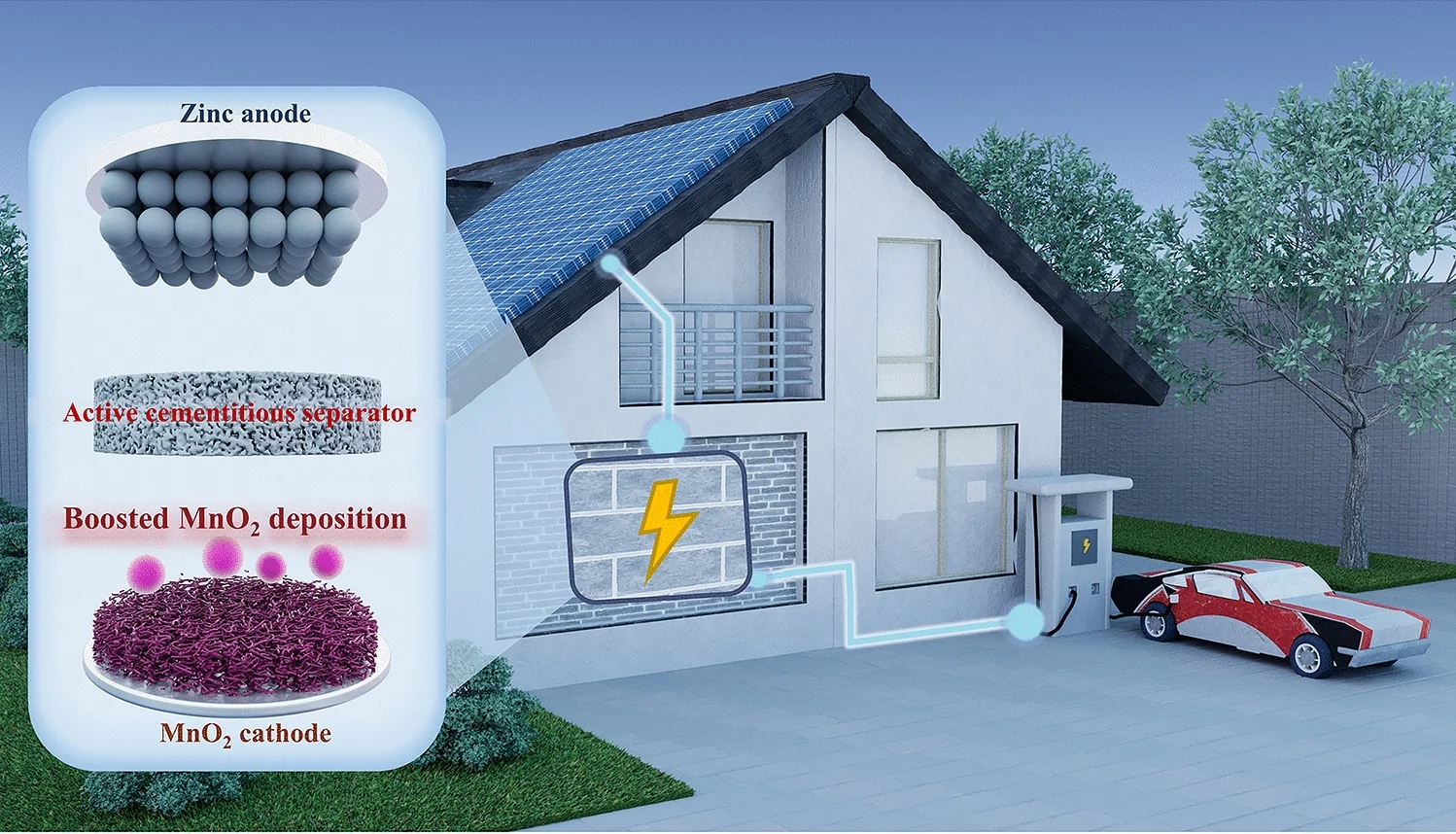
Conceptual schematic of a net-zero energy house assisted by SESSs based on Zn–Mn batteries with ACSs

Inspired by this concept, we present a novel SESS that integrates Zn–Mn batteries with active cementitious separators (ACSs) impregnated with ZnSO_4_ electrolyte and various concentrations of MnSO_4_. As a result, the optimized ACS, with tailored MnSO_4_ concentration, exhibits both enhanced compressive strength (~ 20 MPa) and high ionic conductivity (12.4 mS cm^−1^), enabled by the accelerated side reaction between electrolyte and hydration products. Moreover, the assembled SESS—comprising a zinc foil anode, MnO_2_ cathode and ACSs—exhibits an unprecedented capacity-gaining process, leading to a remarkable specific energy density of 0.92 mWh cm^−2^ at 1.15 mW cm^−2^, a high volumetric energy density of 2.30 kWh m^−3^ at 2.88 kW m^−3^ and exceptional cycling stability with 99.98% capacity retention over 1000 cycles. Mechanistic studies reveal that the zinc sulfate hydroxide (ZSH) formation within the ACSs not only improve the compressive strength but also promotes birnessite-MnO_2_ deposition through H^+^ consumption, driving the observed capacity-gaining and capacity-sustaining behavior. These findings highlight a groundbreaking strategy to endow structural materials with energy storage functionality, enabling the efficient use of renewable energy (e.g., photovoltaics), powering building-scale electricity demands—including household appliances and electric vehicles—and paving the way toward net-zero energy buildings (Fig. 1).

## Results and Discussion

### Preparation and Characterization of ACSs

**Fig. 2:**
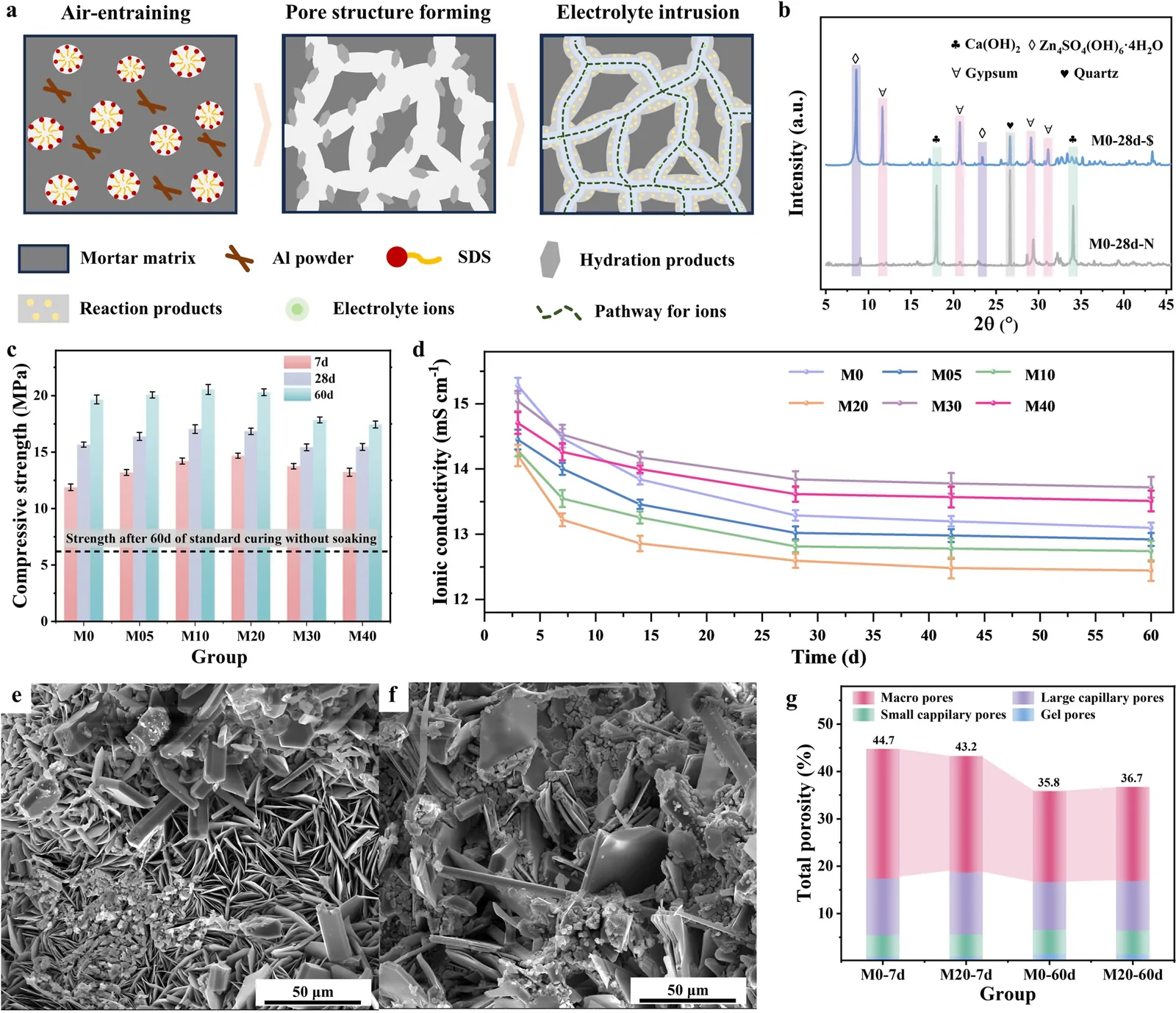
Preparation and characterization of ACSs. **a** Processing of ACSs through air-entraining and vacuum infiltration. **b** XRD patterns of ACSs cured in deionized water for 28d days (marked as “M0-28d-N”) and soaked in 2 M ZnSO_4_ solutions for 28 days (marked as “M0-28d-$”). **c** Compressive strengths of groups soaking in various MnSO_4_ concentration. **d** Ionic conductivity as a function of soaking time for different groups. SEM images of reaction products for **e** “M0-28d” group and **f** “M20-28d” group. **g** Total porosity and distribution of various groups (note: the pore size classifications are detailed in Fig. S6)

As illustrated in Fig. 2a, the cement mortar was aerated using a combined foaming strategy involving aluminum powder (AP) and sodium dodecyl sulfate (SDS), which enables the formation of a highly interconnected pore structure even at low additive dosages [29]. Specifically, AP functions as a gas-generating agent, producing gas through chemical reactions with alkaline constituents in the fresh cement paste [30]. In contrast, SDS acts as a surfactant that promotes stable air-bubble formation during mixing by lowering the interfacial energy and surface tension of the cement paste matrix [31]. Compared with single-agent foaming methods reported previously, this synergistic AP-SDS approach allows more effective pore generation while minimizing adverse impacts on mechanical integrity. To balance mechanical properties and electrochemical performance, AP and SDS were introduced at optimized dosages of 0.1 and 0.015 wt% relative to the cement mass, respectively [8]. Subsequently, the ACS was fabricated via vacuum impregnation of the aerated mortar in sufficient ZnSO_4_-based electrolyte with a liquid-to-solid ratio of 50:1 for a specific duration, ensuring complete filling of the pores with the electrolyte. As shown in Fig. 2b, ZnSO_4_ electrolyte was proved to react with calcium hydroxide (CH), generated from hydration of cement minerals (e.g., (CaO)_3_∙SiO_2_), which was further confirmed by our previous study [8, 32]. Upon soaking, significant amounts of ZSH and gypsum form at the expense of CH. To optimize the intrinsic electrochemical activity of ACSs, the separators were pretreated in electrolytes containing different MnSO_4_ concentrations (0, 0.05, 0.10, 0.20, 0.30 and 0.40 M) dissolved in 2 M ZnSO_4_. The corresponding samples were denoted as M0, M05, M10, M20, M30 and M40, respectively.

As load-bearing components and ion transport media in SESSs, ACSs must exhibit both sufficient mechanical strength and high ionic conductivity. The compressive strength of each group was measured at different soaking durations (Fig. 2c). Overall, electrolyte soaking significantly enhanced compressive strength of ACSs compared to those cured under standard conditions without soaking (6.2 MPa at 60 days). This strengthening effect increased with soaking time. Notably, the presence of MnSO_4_ further accelerated strength development, particularly at early ages. Strength increased with MnSO_4_ concentration, reaching a peak at 0.2 M, but declined slightly at higher concentrations or with prolonged soaking. At 60 days, ACSs with MnSO_4_ concentrations of 0.2 M or lower exhibited compressive strength approaching 20 MPa. The observed decline beyond 0.2 M MnSO_4_ likely due to the retarding effect of Mn^2+^ on cement hydration, which slows strength development over time [33, 34], counteracting the previously reported strengthening effect of ZnSO_4_ soaking [8, 32].

Ionic conductivity, which is closely correlated with compressive strength, followed an inverse trend (Fig. 2d). In general, ACSs with higher compressive strength exhibited lower ionic conductivity. Over time, ionic conductivity decreased rapidly before stabilizing. Groups with MnSO_4_ concentrations below 0.2 M showed a steeper early decline in conductivity, aligning with their rapid strength gain. Interestingly, the M40 group had both lower compressive strength and reduced conductivity compared to M30, likely due to increased electrolyte viscosity at higher MnSO_4_ concentration, which impairs ion mobility (Fig. S1) [35].

To further elucidate the strengthening effect of MnSO_4_, X-ray fluorescence (XRF) analysis was performed. The results confirmed that only trace amounts of Mn were retained in ACSs (Fig. S2), indicating minimal chemical interaction with hydration products. X-ray diffraction (XRD) analysis revealed that all groups predominantly contained ZSH and gypsum (Fig. S3). Notably, MnSO_4_ addition enhanced the formation of these phases at early ages (Figs. S4, S5). Scanning electron microscopy (SEM) images further supported this, showing that in the M20 group, ZSH formed a stacked structure (Fig. 2e), whereas in the M0 group, it appeared as individual layers (Fig. 2f). This suggests that MnSO_4_ facilitate early-age reaction product formation, improving the pore-filling effect, as confirmed by MIP and X-CT (Figs. S6 and S7). The quantified total porosity and pore size distribution (Fig. 2g**)** further explain how MnSO_4_ contributes to early-age strength development.

### Electrochemical Properties of Structural Zn–Mn Batteries with ACSs

**Fig. 3:**
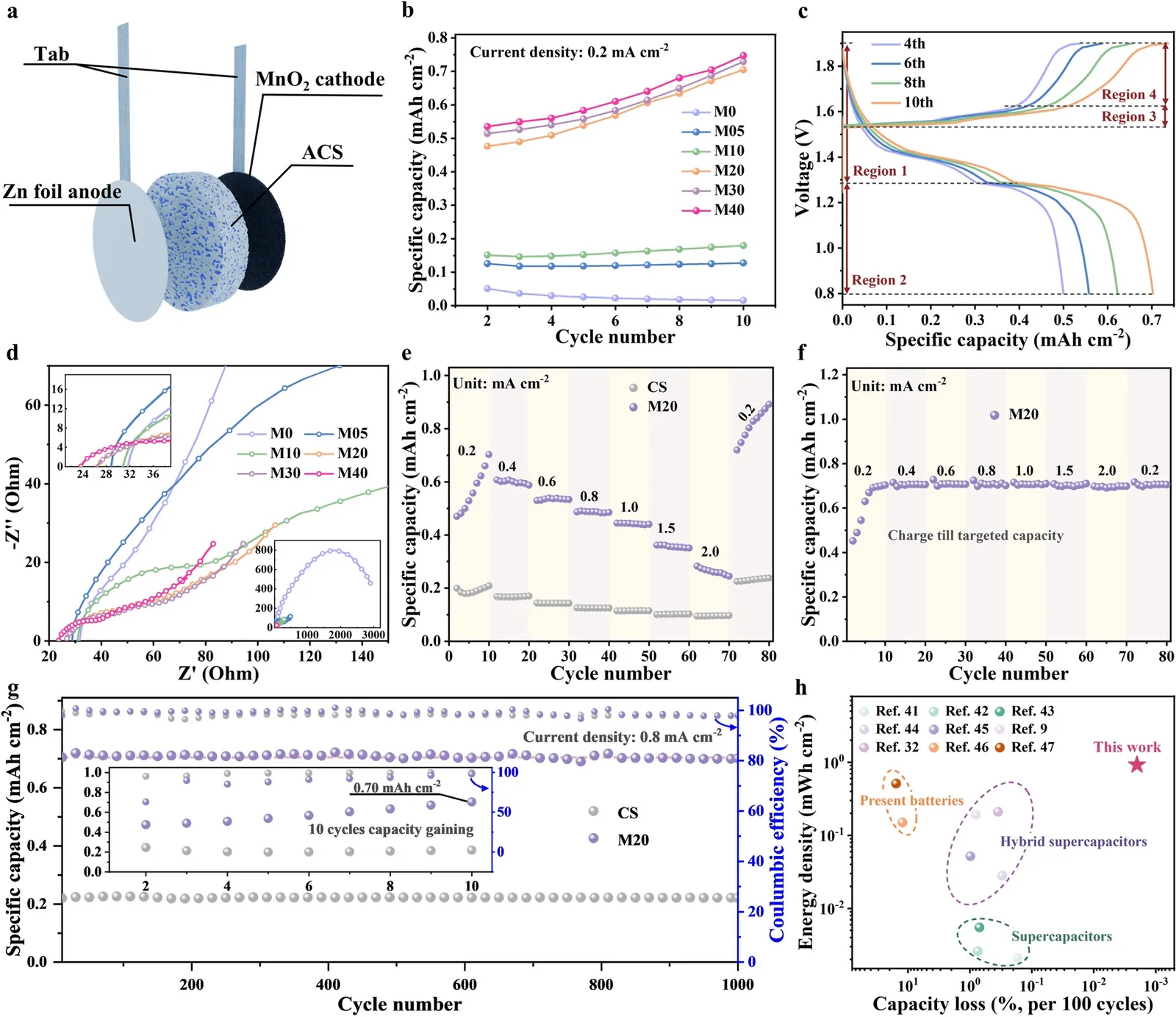
Electrochemical performance of structural Zn–Mn batteries with ACSs. **a** Fabrication of the structural Zn–Mn batteries with ACSs. **b** Evolution of specific capacity during the capacity-gaining process under successive galvanostatic charge–discharge cycles for different groups. **c** Charge–discharge curves within capacity-gaining process for M20 group **d** Nyquist profiles of structural Zn–Mn batteries with ACSs after 10 cycles capacity gaining. **e** Rate performance of structural Zn–Mn batteries with ACSs through galvanostatic charge–discharge. **f** Rate performance of M20 group and CS group with an extra chronoamperometric charge process. **g** Long-term cycling performance of M20 group and CS group with a current density of 0.8 mA cm^−2^. **h** Comparison of this work and related references on energy density and capacity loss rate of structural energy storage devices

Zn–Mn cement-based batteries were fabricated by sandwiching cylindrical ACSs between a commercial zinc foil anode and a MnO_2_ cathode. The assembled battery devices were then hermetically sealed in acrylic enclosures, with the pre-soaked electrolyte added to maintain a stable ionic concentration and to prevent substance exchange with the external environment (Fig. 3a). Further studies on sealing-materials durability and sealing-process optimization are necessary to enhance suitability for practical applications. The adequacy of the electrolyte was evaluated by monitoring its pH evolution, which exhibited a gradually decelerating increase with prolonged soaking time and eventually stabilized at a constant value (Fig. S8). The MnO_2_ used was synthesized via a hydrothermal method (Fig. S9) and identified as α-MnO_2_ (Fig. S10), exhibiting a specific area of 21.7 m^2^ g^−1^ (Fig. S11a) and a rod-like morphology (Fig. S11b). The synthesized α-MnO_2_ was coated onto the stainless steel mesh (SSM) with an optimized loading mass of 1.12 mg cm^−2^ (Fig. S12).

Upon cycling at a current density of 0.2 mA cm^−2^, an unexpected capacity enhancement was observed when the MnSO_4_ concentration reached 0.2 M or higher (Fig. 3b). While increasing MnSO_4_ concentration beyond this threshold continued to accelerate capacity growth, the gain was marginal (0.70 mAh cm^−2^ for M20 group vs. 0.75 mAh cm^−2^ for M40 group after 10 cycles). In contrast, concentrations below 0.1 M resulted either unchanged or slightly degraded capacity. Given these results and the mechanical properties of ACSs, M20 was identified as the optimal composition.

Cyclic voltammetry (CV) further corroborated the capacity enhancement, revealing a significant increase in the enclosed area of the CV curves (Fig. S14). As shown in Fig. 3c, both charge and discharge curves during the capacity-gaining process exhibited two distinct regions, with simultaneous capacity growth in each (Fig. S15). Notably, in comparison with the group utilizing the commercial separator (CS), the ACS-based system exhibits a more distinct charging plateau in the range of 1.8–1.9 V, which is associated with MnO_2_ deposition and accounts for the enhanced capacity observed in ACS-based batteries (Fig. S16). Interestingly, before capacity enhancement, the resistance trends of all groups mirrored their respective ionic conductivities at various MnSO_4_ concentrations (Fig. S17). However, as shown in Fig. 3d, after 10 cycles, the resistance of groups with MnSO_4_ concentrations below 0.1 M remained largely unchanged, whereas those with MnSO_4_ concentrations of 0.2 M or higher showed a marked resistance decrease, highlighting the beneficial role of ACSs in ions transport.

To benchmark ACSs against conventional separators, the electrochemical properties of Zn–Mn batteries with M20 ACSs were compared to those using a CS in the same electrolyte. As shown in Fig. S18, the CS-based battery exhibited a pair of redox peaks at 1.56 and 1.23 V *vs.* Zn/Zn^2+^. In contrast, the M20-based battery displayed significantly higher current and a broader oxidation peak at a higher potential, indicating superior capacity. Rate performance tests conducted at current densities from 0.2 to 2.0 mA cm^−2^ (Fig. 3e) further demonstrated the advantage of ACSs, with M20 exhibiting higher capacity at all current densities due to the additional capacity gain. However, capacity remaining at higher current density is limited. The inherently higher resistance of ACSs may lead to an incomplete charge–discharge process, which causes the formation of electrochemically inactive "dead MnO_2_" [36].

Notably, the capacity gaining of M20 continued to increase with cycling, exceeding 0.82 mAh cm^−2^ under prolonged cycling at 0.2 mA cm^−2^ (Fig. S19). To protect the battery and reserve a fraction of capacity for long-term stability, 85% of the maximum capacity was designated as the operational target. After 10 cycles’ capacity gaining at 0.2 mA cm^−2^, an additional chronoamperometric charging step was introduced to compensate for capacity decline at higher current densities. Specifically, the system was held at a constant potential of 1.9 V until the targeted capacity (0.70 mAh cm^−2^) was recovered. As a result, the M20 group successfully maintained this enhanced capacity even at elevated current densities (Fig. 3f), delivering impressive energy densities (0.94–0.85 mWh cm^−2^) at high power densities ranging from 0.30 to 2.67 mW cm^−2^, highlighting excellent performance robustness across varied power outputs (Fig. S20). What’s more, M20 group achieved a capacity of 0.70 mAh cm^−2^ with a retention rate of 99.98% over 1000 cycles at a current density of 0.8 mA cm^−2^, demonstrating remarkable cycling stability (Fig. 3g). Moreover, even at current densities of 0.2 and 2 mA cm^−2^, the M20 group maintains stable cycling performance without noticeable degradation, indicating its robust compatibility with varying current density conditions (Fig. S21).

Zn||Zn symmetric cell tests revealed that the CS group failed after 300 h due to dendrite-induced short circuits, whereas M20, benefiting from the dendrite-blocking effect of ACSs, maintained stable cycling for 600 h (Fig. S22). This underscores the dual role of ACSs: While they enhance the energy density of cement-based Zn–Mn batteries and suppress short circuits induced by dendrite growth, heterogeneous ZSH formation may accelerate Zn anode degradation [37, 38], necessitating careful regulation in future studies [39, 40]. Additionally, as shown in Fig. S23, the hydrogen evolution potential of M20 was slightly lower than that of CS, suggesting a mitigation of zinc anodes side reaction. The phenomenon may be attributed to the elevated pH value in ACSs-based systems, which will be further explored in Sect. 2.3.

A performance comparison between this study and state-of-art systems is presented in Fig. 3h. Current SESSs predominantly employ conventional supercapacitors and hybrid supercapacitors. Remarkably, our newly developed SESS, which is based on Zn–Mn batteries with the subtle utilization of intrinsic electrochemical activity of cement (ACSs), achieves a ten-fold increase in specific energy density compared to existing technologies, coupled with superior capacity retention over extended cycling periods [8, 32, 41–47]. This substantial improvement establishes a new benchmark for high-performance, durable structural energy storage tailored for infrastructure applications.

### Scalability and Practical Demonstration of Zn–Mn Cement Batteries

**Fig. 4:**
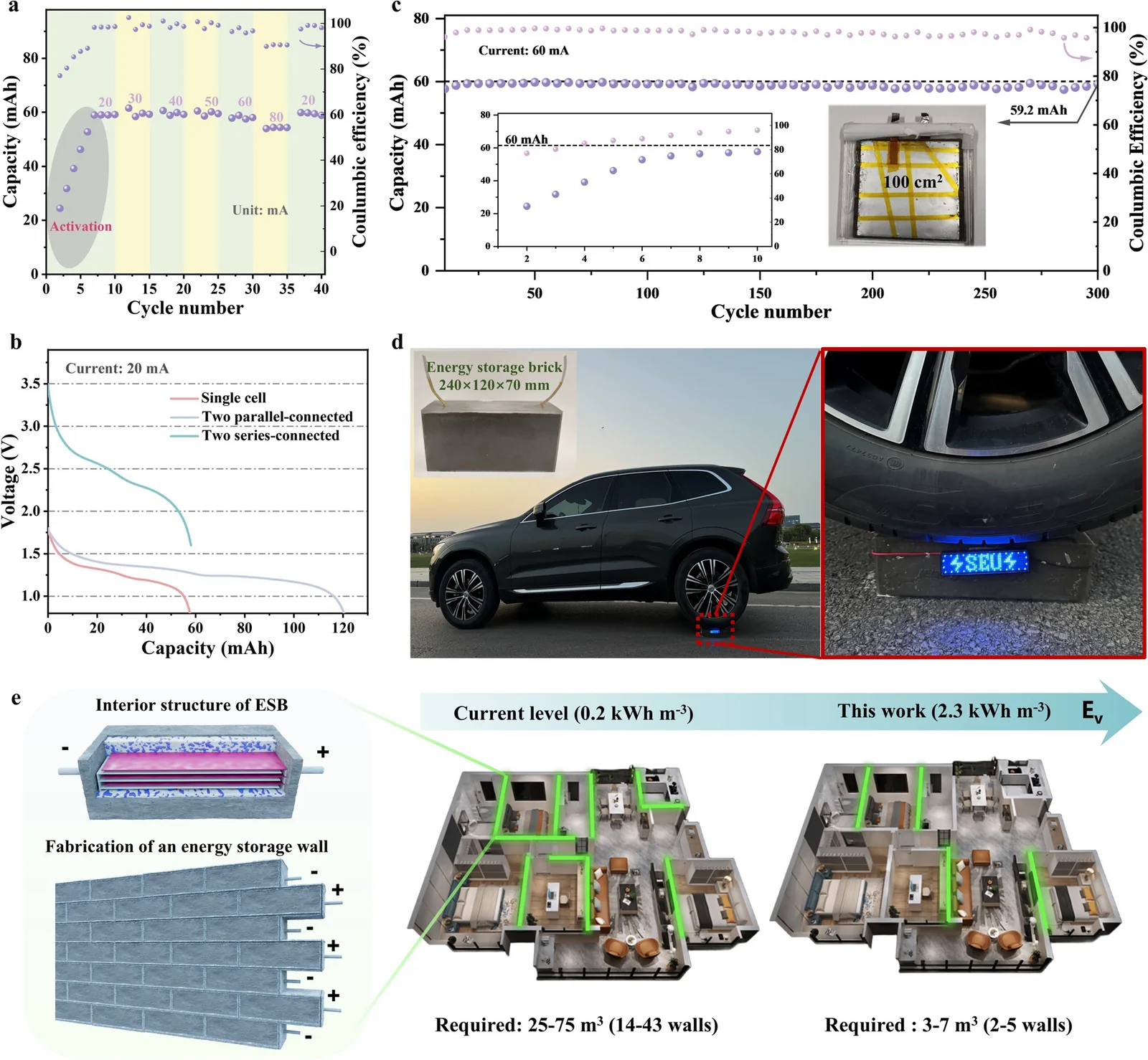
Electrochemical performance and demonstration of the structural Zn–Mn batteries with ACSs at large scale. **a** Rate performance of a single 100 cm^2^ device at currents of 20, 30, 40, 50, 60, and 80 mA. **b** Discharge profile for a single 100 cm^2^ device, two parallel collected 100 cm^2^ devices and two tandemly connected 100 cm^2^ devices at a current of 20 mA. **c** Long-term cycling performance of a single 100 cm^2^ device at a constant discharging current of 60 mA. **d** Photograph of an integrated energy storage brick (ESB) capable of lifting a vehicle while illuminating an LED screen. **e** Fabrication of energy storage walls and comparison of the calculated volume required to power a 130 m^2^ apartment at the current performance level (0.2 kWh m^−3^) and in this work (2.3 kWh m^−3^)

To further demonstrate the scalability and practical viability of Zn–Mn cement batteries, a larger device (100 cm^2^) was fabricated using the same methodology [8] and encapsulated in either plastic pouches or acrylic enclosures. Following 10 cycles of capacity activation, the device achieved a stable capacity of 60 mAh, operating reliably across a current range of 10–80 mA **(**Fig. 4a). When two cells are connected in series, the output voltage doubled (1.6–3.8 V) without scarifying capacity **(**Fig. 4b). In parallel configurations, the discharge capacity increased to 120 mAh, demonstrating effective scalability. Notably, tandemly connected devices successfully powered a light-emitting diode under a 10 kg load (Fig. S24).

Long-term cycling tests revealed a continuously increase in discharge capacity during the initial cycles, stabilizing after 300 cycles (Fig. 4c). To further validate the structural energy storage capability, an integrated energy storage brick (ESB) was fabricated by assembling six electrode sets in parallel. A splitting test was performed to evaluate the interfacial bonding between the electrodes and the cement-based electrolyte, confirming excellent interfacial contact and mechanical integrity (Figs. S25 and S26). The sealed ESB not only powered an LED screen but also supported the weight of a car, simultaneously showing both energy storage performance and mechanical load-bearing capacity (Fig. 4d). These results underscore the dual functionality of Zn–Mn cement batteries, bridging energy storage and structural resilience of scalable applications.

As shown in Fig. 4e, we consider a typical apartment with a floor area of 130 m^2^ and a daily electricity demand of 5–15 kWh. The interior walls (single wall size: 3.0 × 2.4 × 0.24 m^3^) can be constructed using ESBs. At the current performance level [8, 42–45, 48], assuming an energy density of 0.2 kWh m^−3^ for conventional energy storage concrete, 25–75 m^3^ of such material, equivalent to 14–43 walls, would be required to meet the daily electricity demand, exceeding the available volume of interior walls in a typical apartment (approximately nine walls). In contrast, the Zn–Mn cement batteries developed in this work exhibit a markedly higher energy density of 2.3 kWh m^−3^, requiring only 3–7 m^3^ of energy storage concrete—approximately 2–5 walls—to fully power the apartment. This significant reduction in required volume underscores the potential of our approach to turn existing structural elements into effectively energy storage components, advancing zero-carbon buildings.

### Role of ACSs for Enhancing Zn–Mn Cement Batteries

#### Capacity-Gaining Mechanism of Zn–Mn Cement Batteries

To elucidate the role of ACSs in enhancing capacity and improving the electrochemical performance—particularly cycling stability—in Zn–Mn cement batteries, a series of ACSs and their corresponding MnO_2_ cathodes were analyzed after 10, 20, 30, 50 and 100 cycles of capacity gaining. As shown in Fig. S27, brown deposits progressively accumulate on ACS surfaces, increasing with cycle number. Meanwhile, mass measurements of cathodes subjected to different cycles exhibit a strong linear correlation with capacity (Fig. S28), indicating that capacity growth during this process results from the deposition of active materials.

To identify the composition of these deposits, a pristine stainless steel mesh was used as the cathode and subjected to the same capacity-gaining procedure. As expected, a notable capacity enhancement was observed (Fig. S29), yielding an activated cathode with an areal capacity of 0.54 mAh cm^−2^. During the first charge process, the in situ Raman spectroscopy (Fig. S30) revealed the emergence and gradual intensification of three characteristic bands around 510, 580 and 630 cm^−1^. These correspond to the Mn–O stretching vibrations of [MnO_6_] octahedra, basal-plane Mn–O stretching typical of layered manganese oxides and symmetric stretching vibration of the [MnO_6_] group, respectively [26, 49, 50].

SEM imaging coupled with EDS analysis confirmed the formation of Mn- and O-rich nanoflower-like structures (Fig. S31). XRD patterns (Fig. S32) identified the primary phase as birnessite-MnO_2_ (PDF #18-0802), consistent with previous studies reporting that ZSH facilitates birnessite-MnO_2_ deposition in the presence of available Mn^2+^ ions [51, 52]. TEM imaging revealed ultrathin nanosheet, whose elemental composition matched that observed by SEM–EDS (Fig. S33a). Further phase confirmation was achieved via high-resolution TEM (HRTEM) and selected area electron diffraction (SAED), which revealed a lattice fringe spacing of 0.24 nm corresponding to the (006) plane—consistent with both HETEM and XRD findings (Fig. S33b)—thereby validating the formation of birnessite-MnO_2_.

The morphological evolution of ACSs further highlights their role in the capacity-gaining process (Fig. S34). Initially, there was a large amount of ZSH embedded on ACS surfaces. After the 1st discharge cycle, additional ZSH formed. During the 2nd cycle, ZSH partially dissolved upon charging and redeposited upon discharging. After 5 cycles, no residual ZSH remained on ACS surfaces at full charge, suggesting its active involvement in capacity enhancement. Notably, cracks formed due to ZSH dissolution, likely reducing resistance post-capacity gaining. Furthermore, SEM and XRD analyses (Figs. S35 and S36) confirm that ZSH generated on ACS surfaces at full discharge originates from the cathode.

**Fig. 5:**
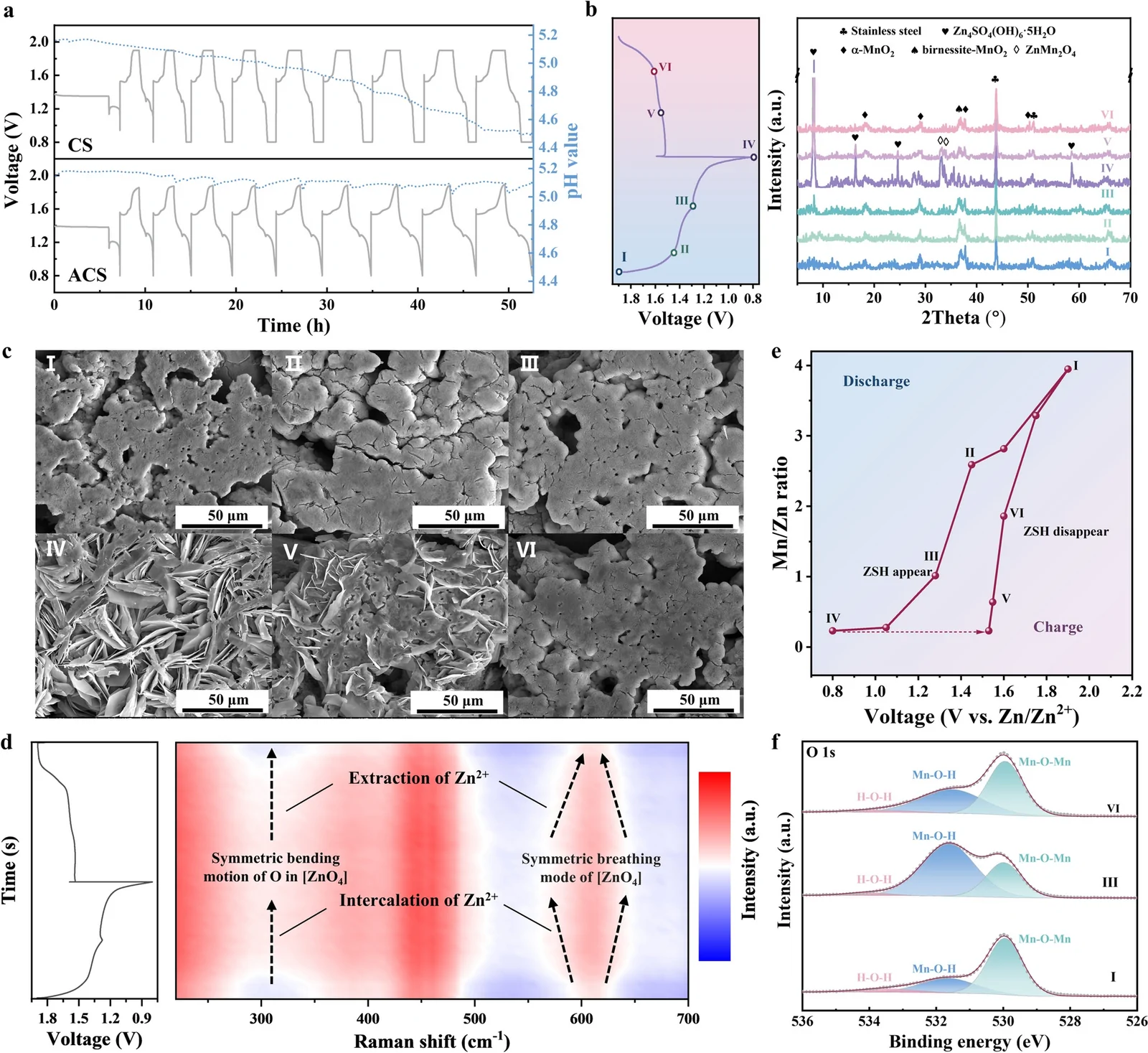
Charge–discharge mechanism of structural Zn–Mn batteries with ACSs. **a** In situ pH monitoring of the M20 and CS groups during the capacity-gaining stage. **b** Ex situ XRD patterns of the cathode surface at corresponding states during charge–discharge process. **c** SEM images of the cathode surface at representative stages (I-VI) during the charge–discharge process of the M20 group after 10 cycles of capacity gaining. **d** In situ Raman spectra of the M20 group during the charge–discharge process after 10 cycles of capacity gaining. **e** Variation of the Mn/Zn ratio on the cathode surface over a full charge–discharge cycle after 10 cycles of capacity gaining. **f** High-resolution O 1*s* XPS spectra of the cathode surface at corresponding states during the charge–discharge process

To further elucidate the role of ACSs in capacity gaining, in situ pH monitoring was performed using a Swagelok cell setup. As shown in Fig. 5a, the initial pH of the electrolyte in the ACS group was 5.16 and maintained relatively stable over 5.02 throughout the capacity-gaining process. In contrast, the control group using CS under the identical conditions (i.e., same electrolyte and charging protocol) failed to sustain comparable discharge durations and exhibited a significant pH drop from 5.16 to 4.51. Compared with pH changes induced by the oxygen evolution reaction (OER), linear sweep voltammetry (LSV) results indicate that the pH variation observed in this work is more likely dominated by proton generation associated with MnO_2_ deposition rather than electrolyte decomposition (Fig. S37*)*. This marked acidification suggests that ACSs effectively buffered the pH by continuously producing ZSH, which neutralized the H^+^ ions generated during the capacity-gaining process, thereby sustaining capacity enhancement.

This proposed mechanism of ACSs-induced capacity enhancement is supported by detailed analysis of the α-MnO_2_ based cathode. During the capacity-gaining process, the fully charged product evolves from α-MnO_2_ to a composite of α-MnO_2_ and birnessite-MnO_2_ (Fig. S38). To elucidate the structural evolution, we analyzed the cathode morphology at different stages of a charge–discharge cycle using XRD (Fig. 5b) and SEM (Fig. 5c). The 10th cycle was selected as a representative stage.

#### Charge–Discharge Mechanism of in Zn–Mn Cement Batteries

At the full charge state (stage I), the cathode primarily consists of amorphous, spherical MnO_2_. During initial discharging (stage II), microcracks and surface defects emerge, accompanied by diminished MnO_2_ diffraction peaks, indicating its partial dissolution [53, 54]. Progressing to stage III, a notable turning point appears in the voltage profile, coinciding with the initial formation of flake-like ZSH, as confirmed by its characteristic XRD peak [55–57]. With further discharge (stage IV), extensive ZSH deposition occurs, ultimately covering the cathode surface at full discharge, where its XRD peak intensifies. Concurrently, ZnMn_2_O_4_ (PDF #24-1133) formation happens, signifying Zn^2+^ intercalation into the MnO_2_ structure [58, 59].

During subsequent galvanostatic charging, a distinct plateau is observed (stage V), corresponding to ZSH dissolution, as evidenced by diminished XRD intensity and substantial reduction in flake-like structures. As charging beyond the plateau, both ZSH and ZnMn_2_O_4_ disappear, indicating full Zn^2+^ extraction and ZSH dissolution. At 1.9 V, α-MnO_2_ and birnessite-MnO_2_ regenerate, as evidenced by recovering XRD intensities, thus restoring the initial charged state. Moreover, as shown in Fig. 5d, compared with the fully charged state, the in situ Raman spectra at lower voltage exhibit an enhanced Raman bond at 300 cm^−1^, corresponding to the symmetric bending mode of O in [ZnO_4_], together with a broader Raman bond at 630 cm^−1^, assigned to the symmetric breathing mode of [ZnO_4_]. These spectral features are attributed to the formation of ZnMn_2_O_4_ [55]. Moreover, the shift in Mn/Zn ratio, quantified via SEM/EDS, also provides complementary evidence confirming the reversible Zn^2+^ insertion/extraction, ZSH deposition/dissolution and corresponding MnO_2_ deposition/dissolution (Fig. 5e).

This deposition/dissolution process is closely linked to H^+^ consumption and generation, verified by the high-resolution O 1*s* XPS spectra (Fig. 5f). The O 1*s* spectra reveal three distinct contributions: Mn–O–Mn, Mn–O–H and H–O–H [55, 60]. To avoid potential signal interference from ZSH in the O 1*s* XPS spectra [61], three representative states (Stage Ⅰ, Stage Ⅲ, Stage Ⅵ) with negligible ZSH content were selected for XPS analysis. From stage Ⅰ to Ⅲ, the Mn–O–Mn signal decreases while Mn–O–H increases, indicating H^+^-induced MnO_2_ transformation and the formation of H_*x*_MnO_2_ [62, 63]. In stage Ⅵ, the Mn–O–H contribution diminishes relative to stage Ⅲ but remains higher than in stage Ⅰ, confirming progressive H^+^ extraction from H_*x*_MnO_2_ during charging.

### Energy Storage Mechanism of Zn–Mn Cement Batteries

**Fig. 6:**
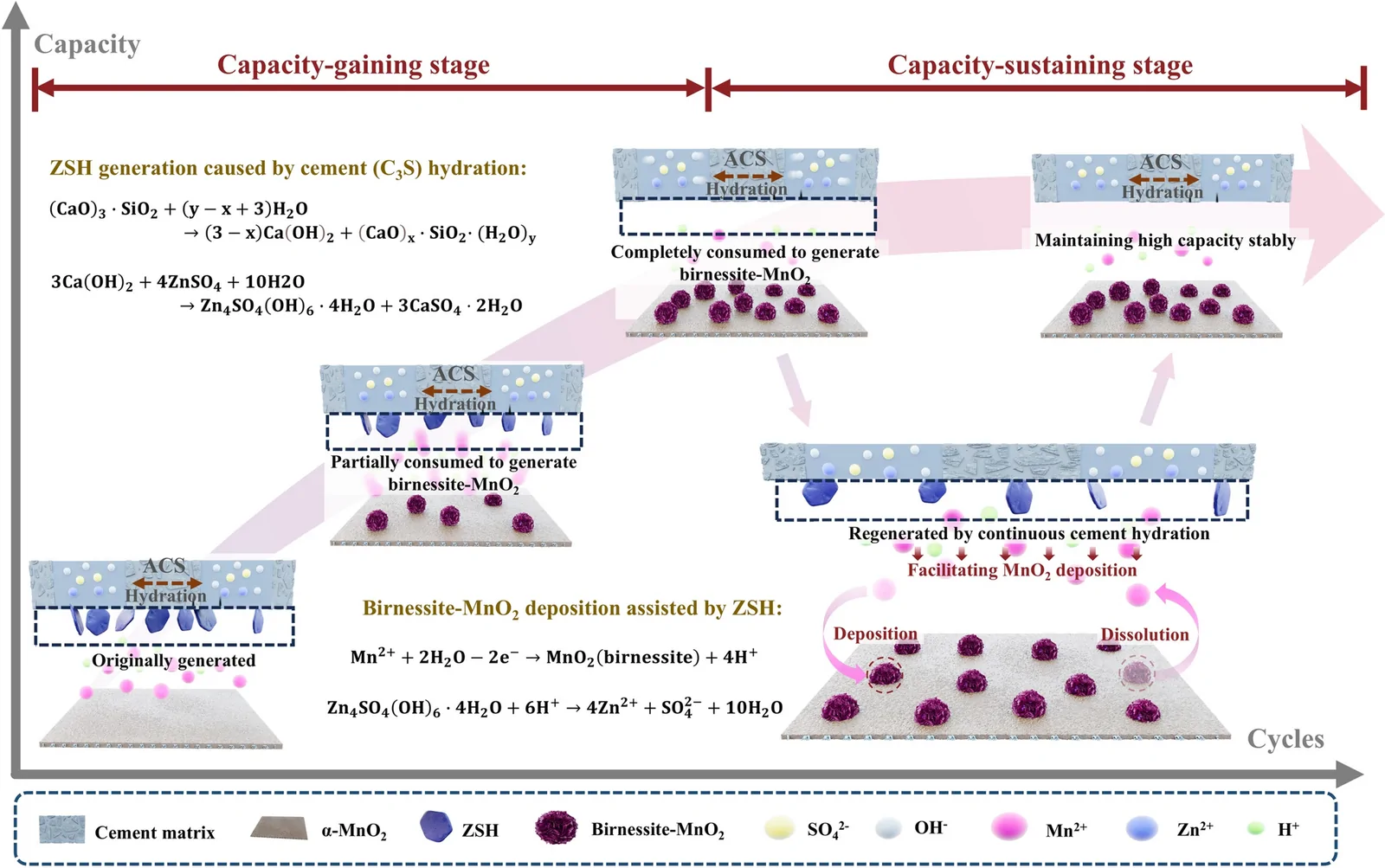
Schematic diagram of the energy storage mechanism for the structural Zn–Mn batteries with ACSs

The energy storage mechanism of structural Zn–Mn batteries incorporating ACSs is illustrated in Fig. 6. The indispensable role of cement arises from its inherently alkaline environment, which sustains reactions between cement hydration products and ZnSO_4_/MnSO_4_ electrolyte. Specifically, before electrolyte soaking (reaction (1)), the hydration of cement minerals (e.g., (CaO)_3_∙SiO_2_) generates significant amounts of Ca(OH)_2_ (CH). Upon contact with ZnSO_4_, CH transforms into ZSH and gypsum (reaction (2)). Owing to the low MnSO_4_ concentration, no Mn-containing precipitates are detected.

During charging, Mn^2^⁺ ions in the electrolyte are oxidized on the cathode and form birnessite-MnO₂. This reaction (reaction (3)) is intrinsically acidic, releasing H⁺ into the local environment. The ZSH generated in reaction (1), acts as a powerful PH buffer (Fig. 5a), consuming H^+^ through reaction (4), thus ensuring the sustained MnO_2_ deposition [25, 64]. While in a conventional neutral ZnSO₄ + MnSO₄ aqueous electrolyte without this cement/ZSH system, the deposition of MnO₂ from Mn^2^⁺ (reaction (3)) is severely hindered and often fails to occur sustainably, because the generated protons (H^+^) rapidly acidify the local environment around cathode make the MnO_2_ deposition (reaction (3)) thermodynamically unfavorable and can even dissolve MnO_2_. Importantly, this reaction also maintains charge balance because SO_4_^2−^ retained in ACSs is counterbalanced by gypsum (CaSO_4_∙2H_2_O) formation. This mechanistic pathway explains why such capacity enhancement occurs only when ACS are used in direct contact with electrodes; no improvement is observed when CSs are employed or when the ACS are isolated from the electrodes in the electrolyte (Fig. S40).

During the capacity-sustaining stage, ZSH continues to nucleate on both the electrode and ACS surfaces owing to ongoing cement hydration. This nucleation facilitates further MnO_2_ deposition (reaction (3)) [36]. Consequently, the buffering effect of ZSH reinforces long-term electrochemical stability, helping maintain the high capacity achieved during the capacity-gaining phase.

For a certain discharge cycle, H^+^ and Zn^2+^ insert into MnO_2_ lattice (including initial α-MnO_2_ and in situ deposited binessite-MnO_2_), forming ZnMn_2_O_4_ and H_x_MnO_2_. As a result, ZSH precipitate at the cathode-ACSs interface due to the H^+^ consumption. Upon charging, H^+^ and Zn^2+^ are extracted from ZnMn_2_O_4_ and H_x_MnO_2_, regenerating MnO_2_. Simultaneously, Mn^2+^ released from ACSs deposits on the cathodes as birnessite-MnO_2_ with a substantial increase in the Mn/Zn ratio (reaction (3)), which is the primary driver of capacity growth and capacity stability as mentioned at the beginning of this section.

Generation of ZSH in ACSs:

1 $$ \begin{array}{*{20}c} {\left( {{\mathrm{CaO}}} \right)_{3} \cdot {\mathrm{SiO}}_{2} + \left( {y - x + 3} \right){\mathrm{H}}_{2} {\mathrm{O}} \to \left( {3 - x} \right){\mathrm{Ca}}\left( {{\mathrm{OH}}} \right)_{2} + \left( {{\mathrm{CaO}}} \right)_{x} \cdot {\mathrm{SiO}}_{2} \cdot ({\mathrm{H}}_{2} {\mathrm{O}})_{y} } \\ \end{array} $$

2 $$ \begin{array}{*{20}c} {3{\mathrm{Ca}}\left( {{\mathrm{OH}}} \right)_{2} + 4Z{\mathrm{nSO}}_{4} + 10{\mathrm{H}}_{2} O \to {\mathrm{Zn}}_{4} S{\mathrm{O}}_{4} \left( {{\mathrm{OH}}} \right)_{6} \cdot 4{\mathrm{H}}_{2} {\mathrm{O}} + 3{\mathrm{CaSO}}_{4} \cdot 2{\mathrm{H}}_{2} {\mathrm{O}}} \\ \end{array} $$

Birnessite-MnO_2_ deposition facilitated by ZSH in ACSs:

3 $$ \begin{array}{*{20}c} {{\mathrm{Mn}}^{2 + } + 2{\mathrm{H}}_{2} {\mathrm{O}} - 2{\mathrm{e}}^{ - } \to {\mathrm{MnO}}_{2} \left( {birnessite} \right) + 4{\mathrm{H}}^{ + } } \\ \end{array} $$

4 $$ \begin{array}{*{20}c} {{\mathrm{Zn}}_{4} {\mathrm{SO}}_{4} \left( {{\mathrm{OH}}} \right)_{6} \cdot 4{\mathrm{H}}_{2} {\mathrm{O}} + 6{\mathrm{H}}^{ + } \to 4{\mathrm{Zn}}^{2 + } + {\mathrm{SO}}_{4}^{2 - } + 10{\mathrm{H}}_{2} {\mathrm{O}}} \\ \end{array} $$

Although pH-buffering additives have been explored as a way to improve Zn–Mn batteries [25, 64], the underlying strengthening mechanism remains debated. In our system, cement acts as a vast reservoir, continuously generating ZSH in quantities far exceeding those attainable in conventional Zn–Mn batteries. This abundant ZSH supply accelerate birnessite-MnO_2_ deposition through proton consumption, thereby driving the pronounced capacity enhancement observed here.

## Conclusions

In summary, neutral Zn–Mn batteries are firstly introduced to build SESSs through sandwiching zinc anode, α-MnO_2_ cathode and ACSs that are vacuum impregnated with ZnSO_4_ electrolyte and optimized MnSO_4_ additive. The optimized ACSs exhibit improved compressive strength (~ 20 MPa) and high ionic conductivity (12.4 mS cm^−1^) due to the acceleration of the adverse reaction between electrolyte and hydration products. Subsequently, the assembled SESSs exhibit impressive capacity-gaining process with rapid capacity obtaining, appearing high specific energy density (0.92 mWh cm^−2^ at 1.15 mW cm^−2^), high volumetric energy density (2.30 kWh m^−3^ at 2.88 kW m^−3^) and good cycling stability (99.98% capacity retention after 1000 cycles). Mechanistically, ACSs function as H^+^ buffers via the in situ generation of ZSH from reactions between the ZnSO₄ electrolyte and cement hydration products. This buffering effect has been proved to facilitate the deposition of birnessite-MnO_2_, thereby driving the observed capacity gaining. Beyond ZSH, other alkaline components in the ACSs, particularly the dominant hydration product (C–S–H), provide abundant reactive sites for H^+^ capture, opening further opportunities for capacity enhancement. Moreover, the Mn source can be sourced from industrial waste commonly incorporated into cement. This eliminates the need for soluble Mn^2+^ in the electrolyte and enables the cementitious material itself to function as an active electrochemical component. Consequently, the SESSs based on this Zn–Mn batteries and ACSs integration exhibit outstanding electrochemical properties by converting inert cementitious component into electroactive component. This work highlights a promising strategy for deeply integrating building materials with renewable energy storage, thereby advancing progress toward a zero-carbon future.

## Supplementary Information

Below is the link to the electronic supplementary material.

Supplementary file1 (DOCX 35223 KB)
