# Supplementary material for: High Performance Zn–Mn Cement Batteries for the Next Generation of Buildings
Source: Nanomicro Lett. 2026 Mar 13;18:284. doi: 10.1007/s40820-026-02122-x (PMC12988134; doi:10.1007/s40820-026-02122-x)
Supplement: Supplementary file 1 — Supplementary file1 (DOCX 35223 KB) [file 40820_2026_2122_MOESM1_ESM.docx]

Supporting Information for

**High Performance Zn-Mn Cement Batteries for the Next Generation of Buildings**

Zhaolong Liu^1^, Pan Feng^1,*^, Long Yuan^1^, Ruidan Liu^1^, Xiangyu Meng^1^, Guanghui Tao^1^, Jian Chen^1^, Zaiping Guo^2,*^ and Changwen Miao^1,*^

^1^ State Key Laboratory of Engineering Materials for Major Infrastructure, Southeast University, Nanjing 210008, P. R. China

^2^ School of Chemical Engineering and Advanced Materials, The University of Adelaid, SA 5005, Australia

*Corresponding authors. E-mail: [pan.feng@seu.edu.cn](mailto:pan.feng@seu.edu.cn) (Pan Feng); [zaiping.guo@adelaide.edu.au](mailto:zaiping.guo@adelaide.edu.au) (Zaiping Guo); [mcw@cnjsjk.cn](mailto:mcw@cnjsjk.cn) (Changwen Miao)

# **S1 Supplementary materials and methods**

## S1.1 Materials

Polyvinylidene difluoride (PVDF) was purchased from AKEMA. Acetylene black was purchased from 3A. N-methylpyrrolidone (NMP) and manganese sulfate (MnSO_4_) were purchased from Macklin. Zinc sulfate heptahydrate (ZnSO_4_·7H_2_O) and sodium dodecyl sulfate (SDS) were purchased from Aladdin. All chemicals were of analytical grade and directly used without further purification.

Zn metal foil with a thickness of 100 μm was purchased from Shanghai Weidi Metal Material Co., Ltd. Aluminum powder (AP) with reactive aluminum content at around 90 wt.% was purchased from LIGUANHUAGONG. The cement used in this study was reference cement (P.Ⅰ 42.5) purchased from Qufu Zhonglian Cement Co., Ltd. The sand was ISO standard sand purchased from Xiamen Aisiou Standard Sand Co., Ltd. Ultrapure water was used in all experiments.

## S1.2 Preparation of α-MnO_2_ by hydrothermal method

First, 30 mL of 0.1M KMnO_4_ solution and 30 mL of 0.15 M MnSO_4_ solution were prepared separately and stirred for 1 hour using a magnetic stirrer. The well-stirred KMnO_4_ solution was then added dropwise into the MnSO_4_ solution under continuous stirring, and the mixture was further stirred for another hour. The resulting solution was transferred into a container, sealed in a hydrothermal reactor, and subjected to hydrothermal treatment at 160 ℃ for 12 hours. The MnO_2_ precipitate was collected, thoroughly washed several times with deionized water and anhydrous ethanol, and then dried in an oven at 80 ℃ for 12 hours. Finally, the dried MnO_2_ powder was placed in a tube furnace and calcined at 300 ℃ for 1 hour to obtain α-MnO_2_ powder.

## S1.3 Preparation of α-MnO_2_@SSM cathode

First, 0.35 g of α-MnO_2_, 0.10 g of acetylene black and 0.05 g of PVDF were weighed and thoroughly ground in a mortar. Next, 1.6 mL of NMP was added, and the mixture was stirred continuously for at least 2 minutes to form a uniform slurry. The slurry was then drop-coated onto a stainless steel mesh (SSM, #304 with a mesh size of 74 μm) to form a film with a thickness of approximately 20 μm. The coated mesh was dried in an oven at 60 ℃ for 12 hours, yielding the α-MnO_2_ cathode with an average mass loading of ~1.12 mg cm^-2^.

## S1.4 Preparation of cement-based separators

First, 140 g of ultrapure water was weighed, and 0.03 g of SDS was dissolved in it by continuous stirring. Meanwhile, 200 g of cement and 200 g of sand were blended in a mixing pot. The SDS solution was then added to the dry mixture and stirred slowly for 30 s, followed by rapid mixing for 1.5 min. Subsequently, 0.2 g of AP (0.1 wt% of cement) was added to the slurry and mixed rapidly for an additional 1.5 min.

A portion of the resulting slurry was poured into cubic stainless steel molds (20 mm × 20 mm × 20 mm) for specimens intended for mechanical property testing. The remaining slurry was cast into cylindrical PTFE molds (radius: 12 mm, depth: 4 mm) for electrochemical testing. All specimens with molds were cured for 24 h at 20 ℃ and 90% relative humidity. After curing, excess mortar was trimmed with a knife to ensure a smooth surface.

The specimens were then demolded and dried in a vacuum oven at 45 ℃ for 3 days. Following drying, the specimens were immersed in an electrolyte solution at a liquid-to-solid ratio of 50:1, then subjected to vacuum impregnation. The electrolyte after soaking was subsequently used for electrochemical testing, ensuring consistency between the impregnated ACS and the testing electrolyte.

## S1.5 Measurement of compressive strength

To enhance the efficiency and uniformity of vacuum electrolyte impregnation, specimens with dimensions of 20 mm × 20 mm × 20 mm were used for compressive strength testing after soaking in the electrolyte. Tests were conducted using UTM5105 microcomputer-controlled electronic universal testing machine at a loading rate of 0.5 mm min^-1^. The maximum load *P*_max_ was recorded and used to calculate the compressive strength *CS* (MPa) according to:

$$CS=\frac{P_{\max}}{A_{cub}}$$

where *P*_max_ is the maximum load applied during the test (N), and *A*_cub_ is the loaded surface area of the specimen (mm^2^). In this study, *A*_cub_ =40 mm^2^. Each test was repeated three times per group, and the reported compressive strength is the average of the three measurements.

## S1.6 Measurement of ionic conductivity

Cylinder mortar specimens were used to measure ionic conductivity. Before testing, the cement-based specimen (served as a separator) was positioned between two stainless steel foils, each 20 μm thick. Electrochemical impedance spectroscopy (EIS) measurements were conducted on the assembled device under open-circuit voltage conditions. The recorded data were used to calculate the ionic conductivity *IC* (mS cm^-1^) using the equation:

$$IC=\frac{1000d_{\mathrm{cy}}}{R_{s}A_{\mathrm{ss}}}$$

where *d*_cy_ is the thickness of the cement-based separator (cm), *R*_s_ is the solution resistance (Ω), and *A*_ss_ is the surface area of the stainless steel foil (cm^2^). In this study, *d*_cy_=0.4 cm and *A*_ss_ =1.13 cm^2^. Each test was repeated three times per group, and the reported values represent the average of the three measurements.

## S1.7 Fabrication of structural energy storage devices

First, the zinc foil anode and α-MnO_2_@SSM cathode were each connected to tabs using conductive tape. A cement-based separator was placed between the two electrodes, after which the assembled electrodes and separator were hermetically sealed in an acrylic enclosure with the pre-soaked electrolyte at a liquid-to-solid ratio of 50:1.

## S1.8 Preparation of 100 cm^2^ energy storage devices

An 11 cm × 11 cm × 0.5 cm cement-based separator containing 0.1 wt.% AP was prepared following the same procedure mentioned above. Both the α-MnO_2_@SSM cathode and zinc foil anode had an effective area of 10 cm × 10 cm, with extended sections serving as current tabs. For visual observation, the assembled device was sealed in a 12 cm × 12 cm × 0.8 cm acrylic enclosure using self-adhesive silicone waterstop. Alternatively, for experiments requiring direct force transmission, the pre-assembled device was encapsulated in polyolefin shrink film using a heat-sealing process.

## S1.9 Preparation of energy storage bricks

An in-situ cast aerated cement-based brick (24 cm × 12 cm × 7 cm) was prepared following the procedure mentioned above. The electrodes were identical to those in Section S**1. 8**. After positioning and fixing the electrodes, fresh aerated cement mortar was poured into the mold, filling the spaces between electrodes. The assembled energy storage brick was then sealed with epoxy resin on all sides except the upper surface, allowing for electrolyte infiltration. Following a 14-day vacuum curing in electrolyte, the upper surface was finally sealed by anti-salt glue.

# **S2 Supplementary Figures**

**Fig. S1** Ionic conductivity and pH of 2M ZnSO_4_ solution with different MnSO_4_ concentrations

**Fig. S2** Element components for different groups via XRF

**Fig. S3** XRD patterns of cement-based separators soaked in ZnSO_4_ solutions with varying MnSO_4_ concentrations

**Fig. S4** Semi-quantitative XRD analysis results of the “M0-7d” and “M20-7d” groups


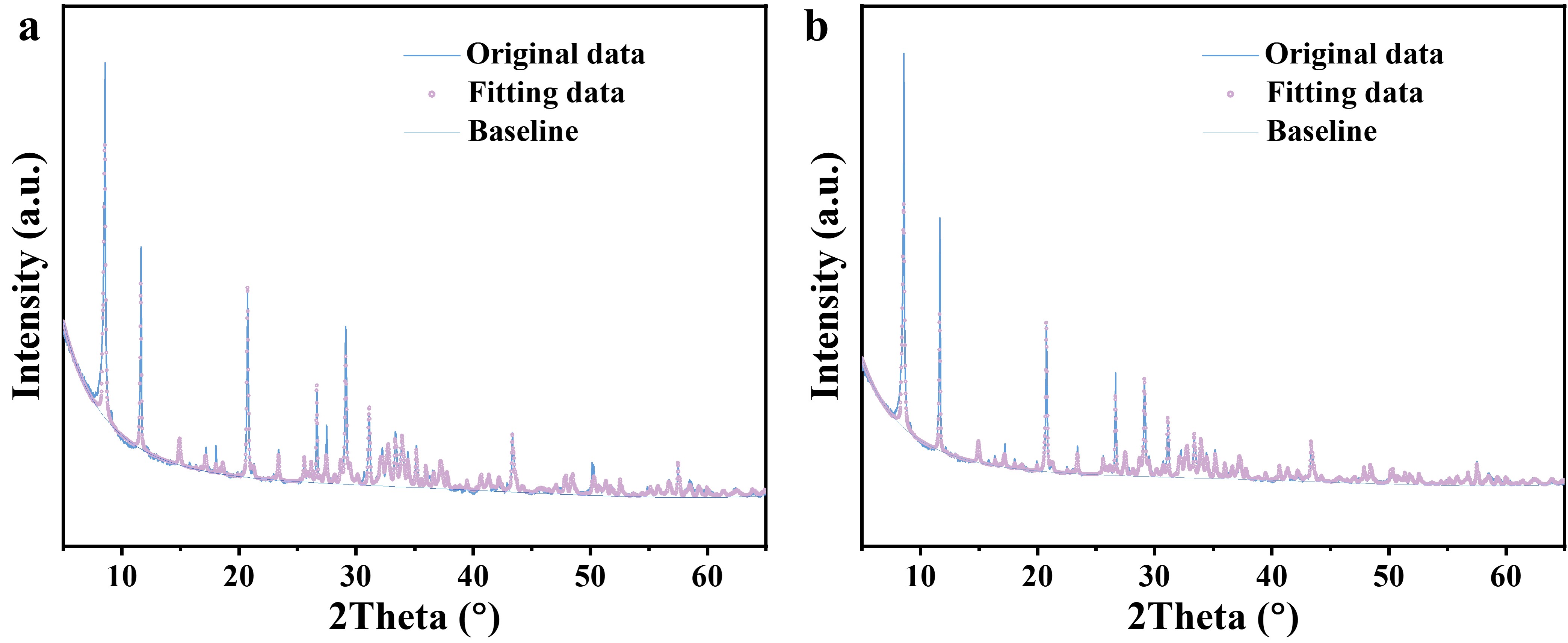


**Fig. S5** XRD fitting profiles of **a** the “M0-7d” group and **b** the “M20-7d” group


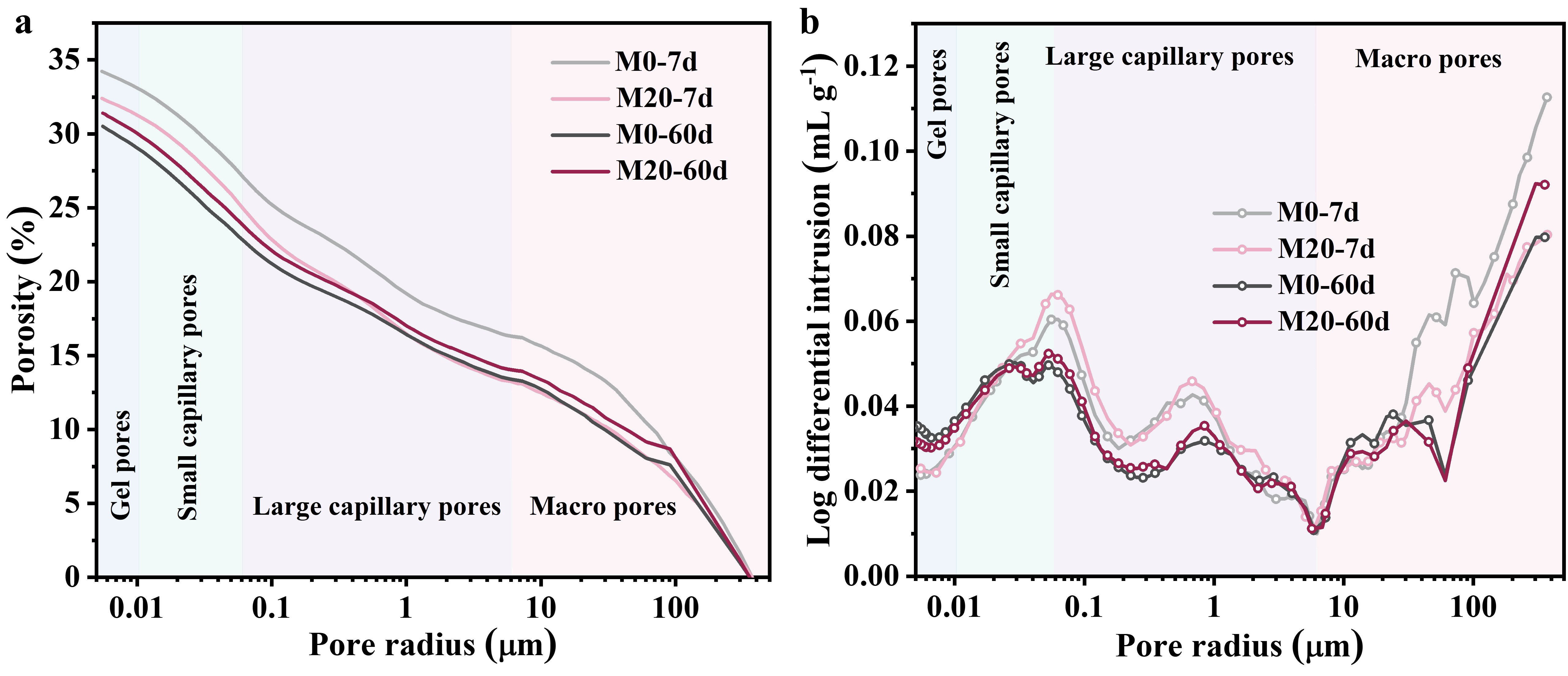


**Fig. S6** Pore size distribution of various groups measured by MIP. **a** Cumulative porosity curves. **b** Log-differential intrusion curves. Pores are categorized as gel pores (0.005-0.01μm), small capillary pores (0.01-0.05μm), large capillary pores (0.05-5μm) and macro pores (>5μm)


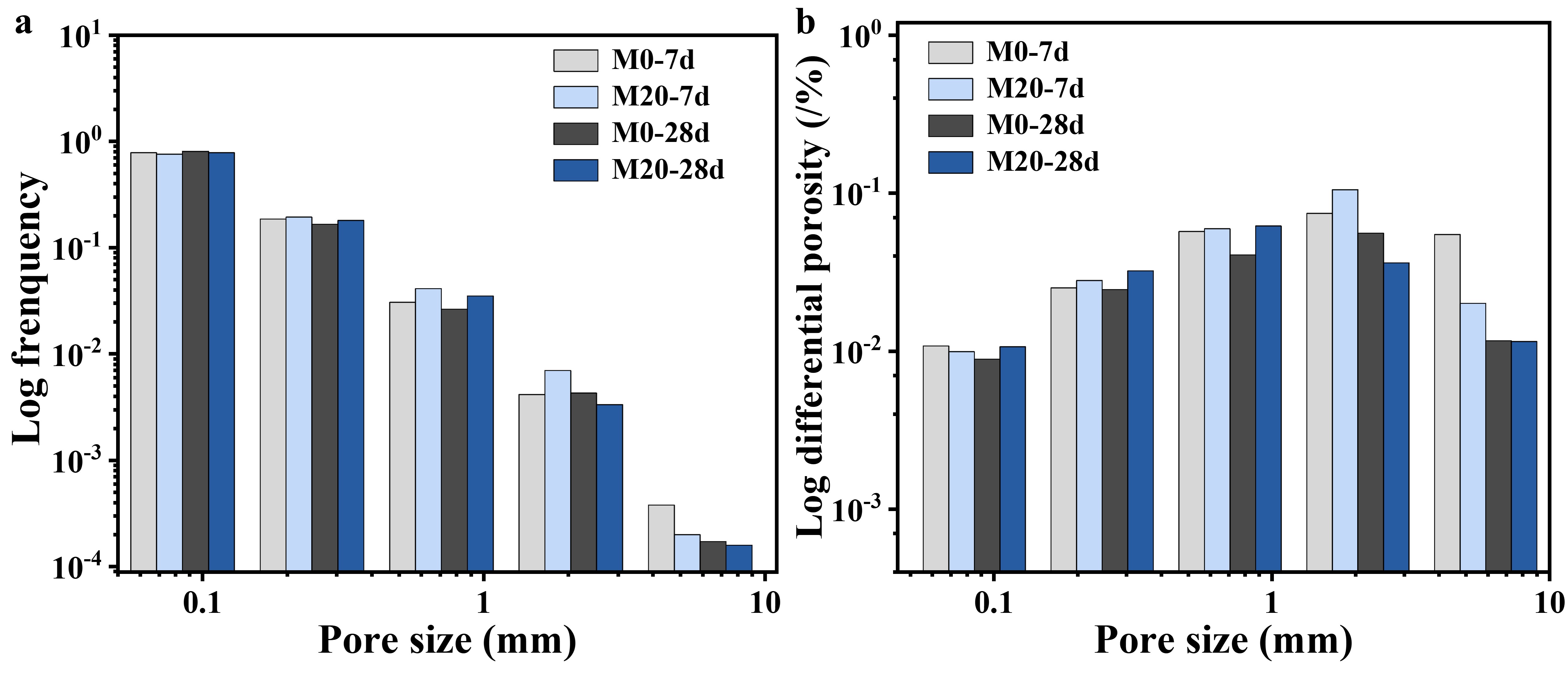


**Fig. S7** Statistical analysis of pore size distribution in various groups via X-CT. **a** Frequency distribution of pore sizes. **b** Porosity distribution of different pore sizes

**Fig. S8** Evolution of electrolyte pH with soaking time


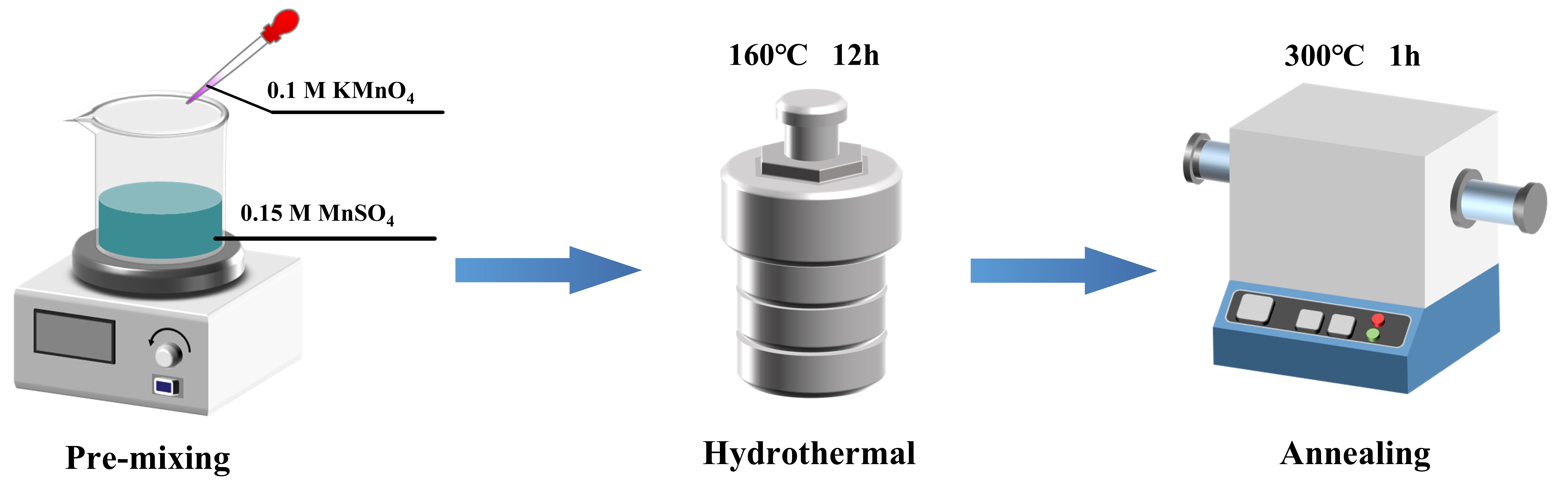


**Fig. S9** Synthesis process of α-MnO_2_ via hydrothermal method

**Fig. S10** XRD pattern of the synthesized α-MnO_2_


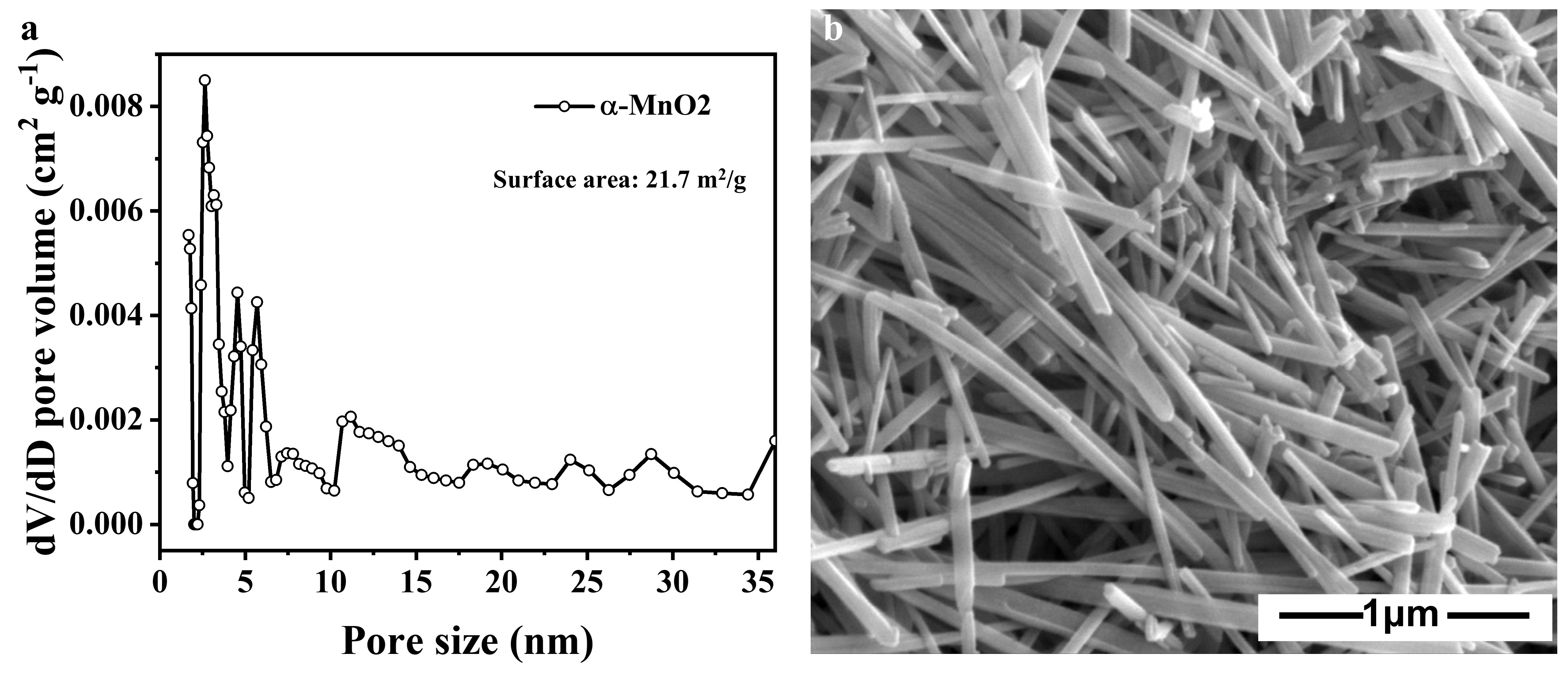


**Fig. S11** Characterization of the synthesized α-MnO_2_. **a** Pore size distribution derived from N_2_ adsorption-desorption isotherms, with a measured surface area of 21.7m^2^ g^-1^. **b** Morphology observed by SEM

**Fig. S12** Evolution of specific capacity during the capacity gaining process with different initial MnO_2_ loadings

As the loading mass increases, the areal capacity increases accordingly whereas the specific capacity decreases. To balance cost-effectiveness and material utilization efficiency, a loading of 1.12 mg cm^-2^ was ultimately chosen as the optimal condition for electrochemical performance characterization.

**Fig. S13** Specific capacity evolution during the capacity gaining process for ACSs subjected to different soaking times

With prolonged soaking time, the gained capacity of structural battery within 10 cycles increases gradually, while the growth rate slows and eventually stabilizes. Based on a balance between electrochemical performance and experimental efficiency, a soaking time of 14 days was selected for all samples.

**Fig. S14** CV curves of different groups using various separators at a scan rate of 1 mV s^-1^

The redox peaks in the CV curves exhibit apparent shifts and distortions at higher scan rates, which can be attributed to severe polarization effects caused by the relatively high internal resistance of the ACSs.


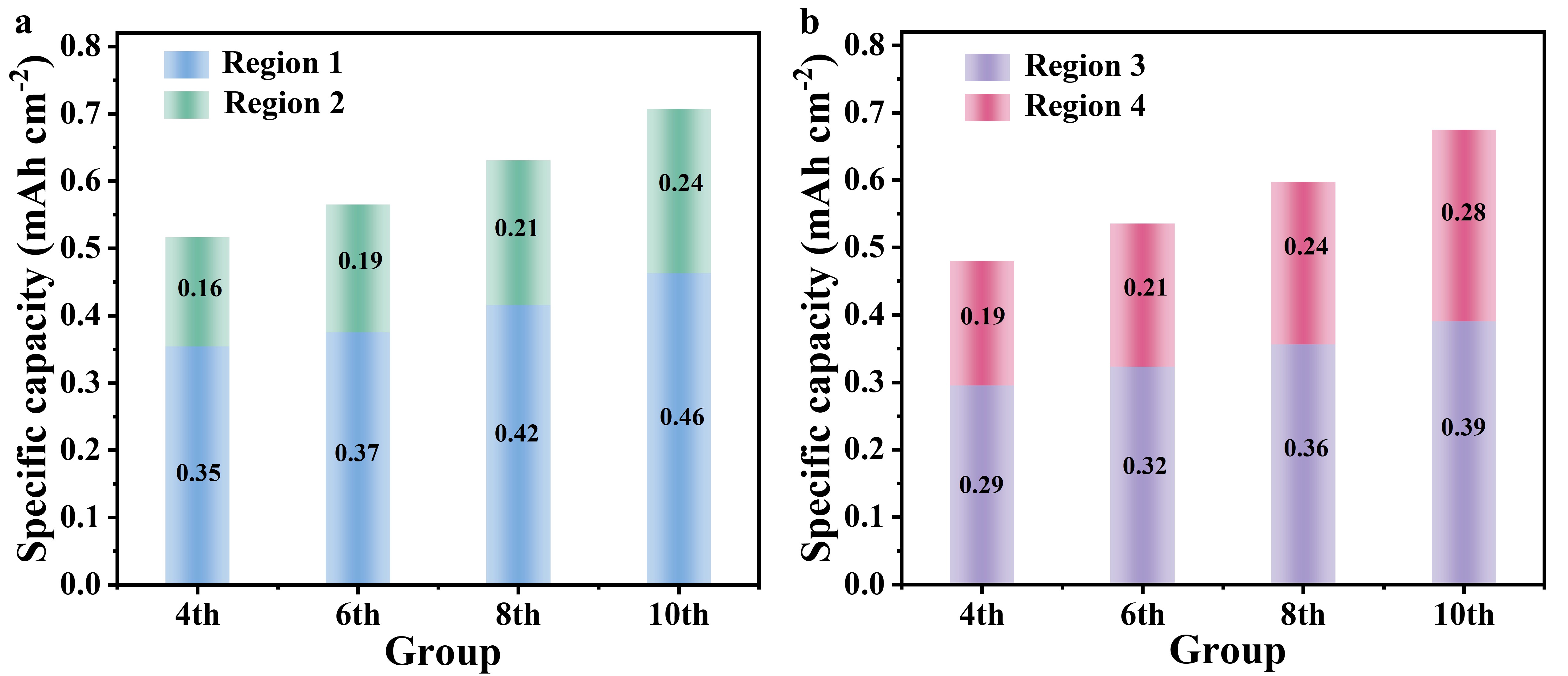


**Fig. S15** Specific capacity contributions of different regions at various activation cycles during **a** the discharge stage and **b** the charge stage

**Fig. S16** Comparison of charge/discharge curves of M20 group and CS group at the 10^th^ cycle

**Fig. S17** Nyquist profiles of structural Zn-Mn batteries with different MnSO_4_ concentrations before activation

**Fig. S18** Comparation of CV curves for CS group and M20 group at a scan rate of 0.1 mV s^-1^

**Fig. S19** Specific capacity of the M20 group with continuous cycling at 0.2 A g^-1^

^^

**Fig. S20** Energy densities versus power densities for the M20 group


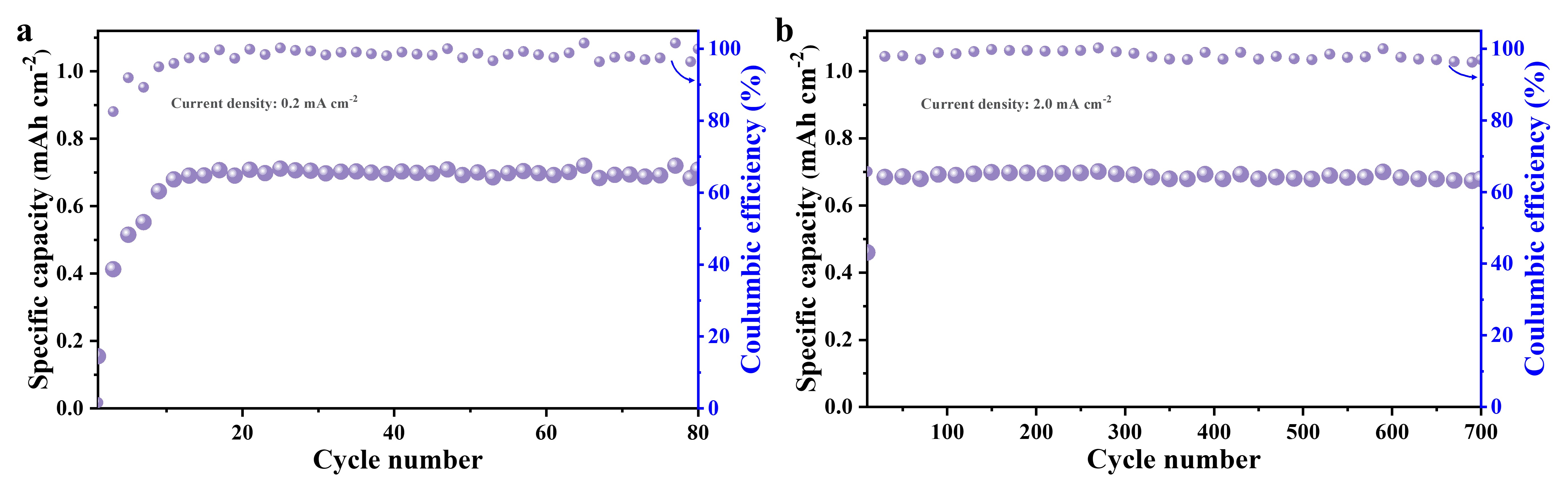


**Fig. S21** Long-term cycling performance of M20 group at **a** 0.2 mA cm^-2^ and at **b** 2 mA cm^-2^

**Fig. S22** Polarization voltage during Zn plating/stripping at a current density of 1.0 mA cm^-2^ and a capacity of 0.5 mAh cm^-2^

**Fig. S23** LSV curves of the CS and M20 groups


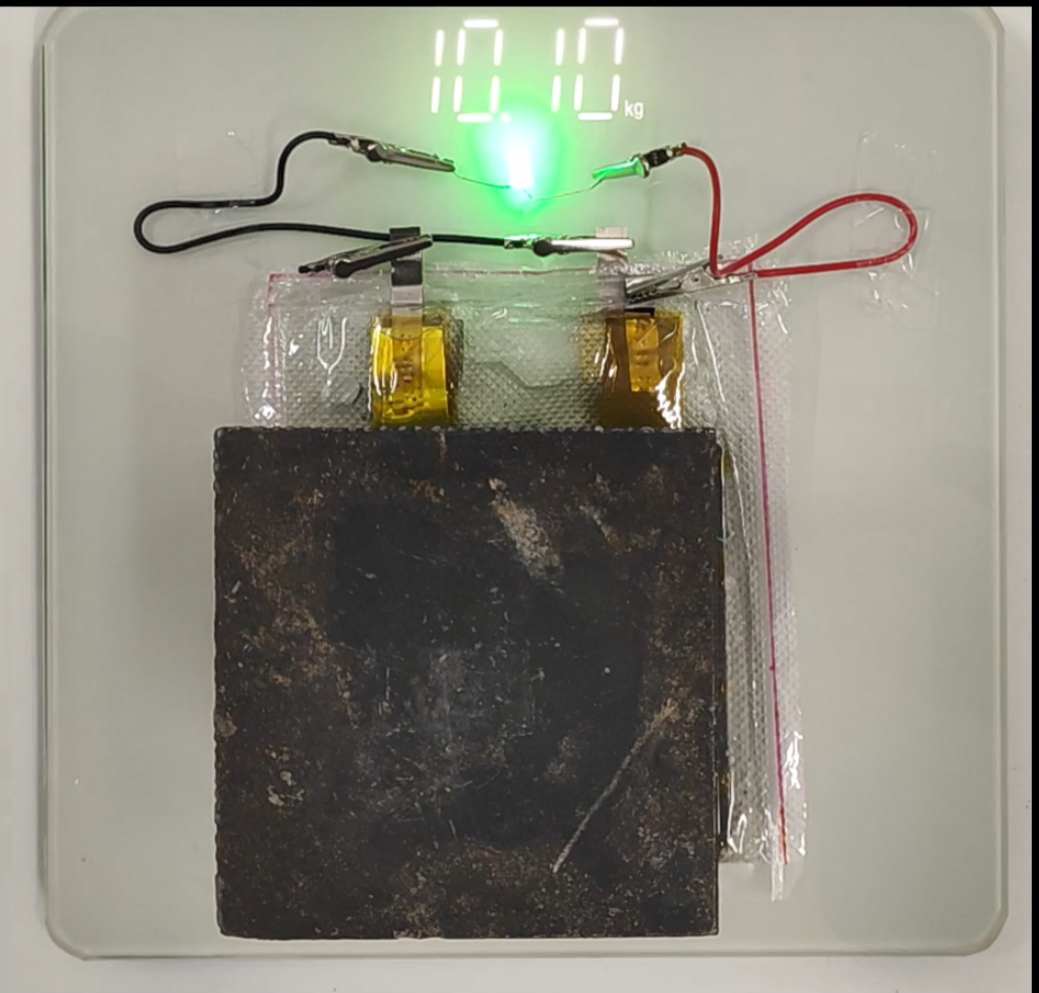


**Fig. S24** Photograph of two tandemly connected 100 cm^2^ devices illuminating a green light-emitting diode under compressive load


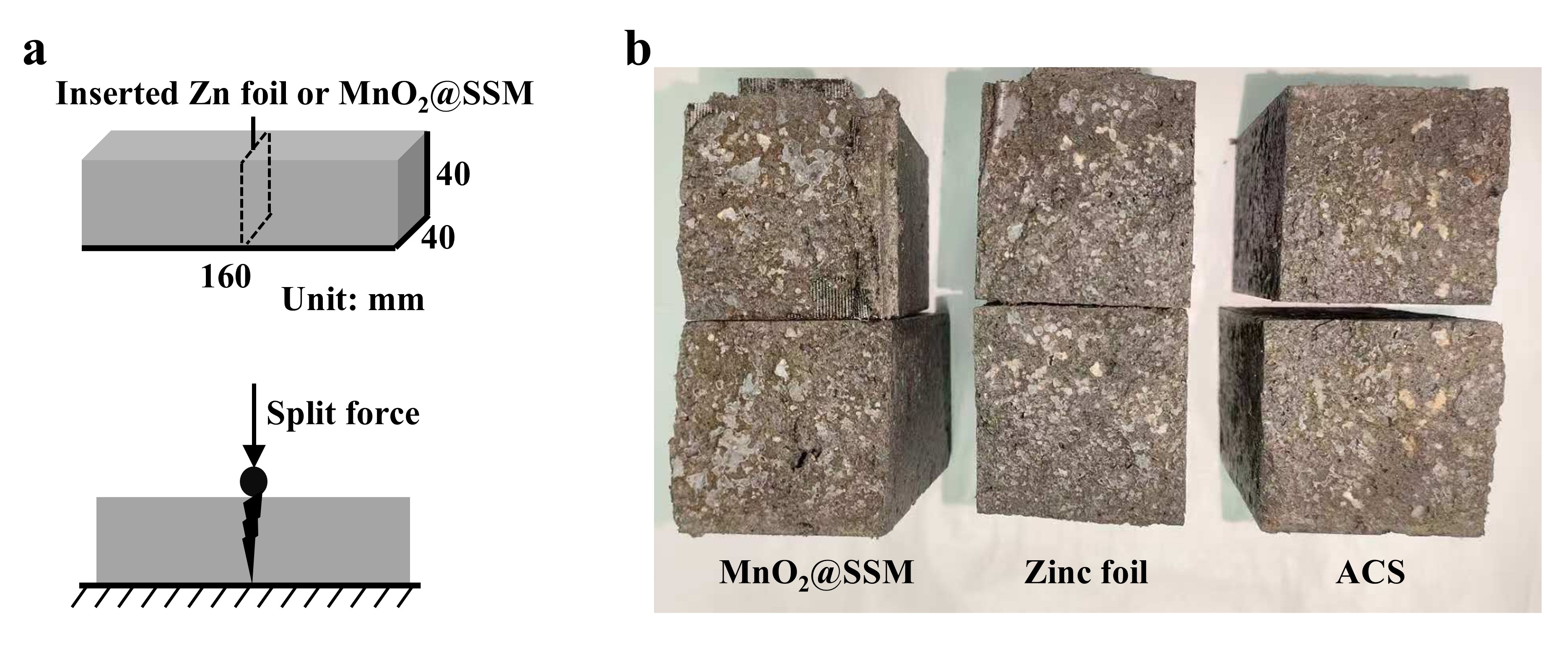


**Fig. S25** **a** Schematic diagram of splitting test. **b** Schematic diagram of the specimens cross-section after splitting

**Fig. S26** Splitting force of specimens containing different electrodes


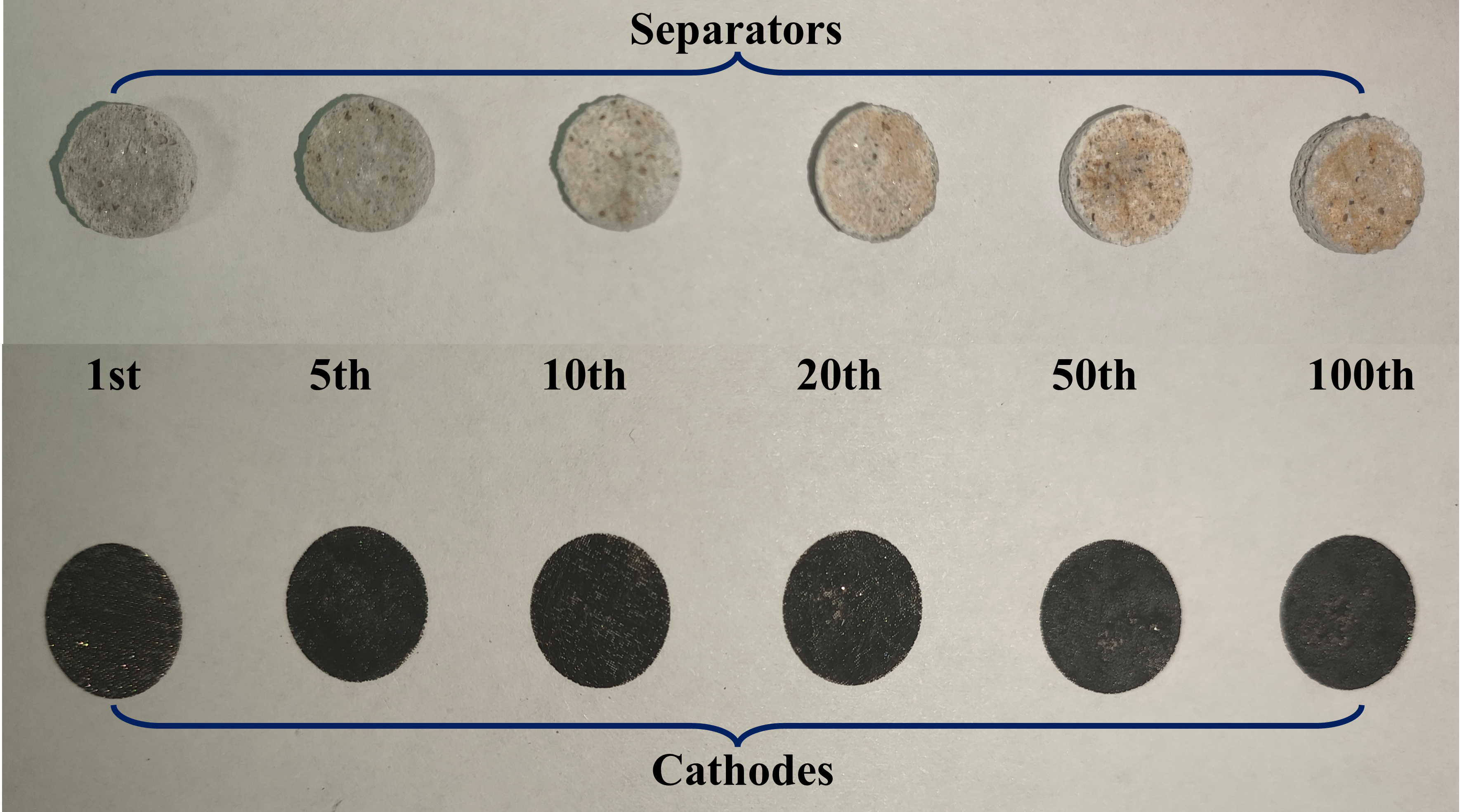


**Fig. S27** Photography of cathodes and ACMs after different activation cycles

**Fig. S28** Fitted relationship between capacity and cathode mass obtained across different activation cycles

**Fig. S29** Evolution of areal capacity of the M20 group with an SSM cathode during the activation process

**Fig. S30** In-situ Raman spectra of the M20 group with an SSM cathode during the first charging process


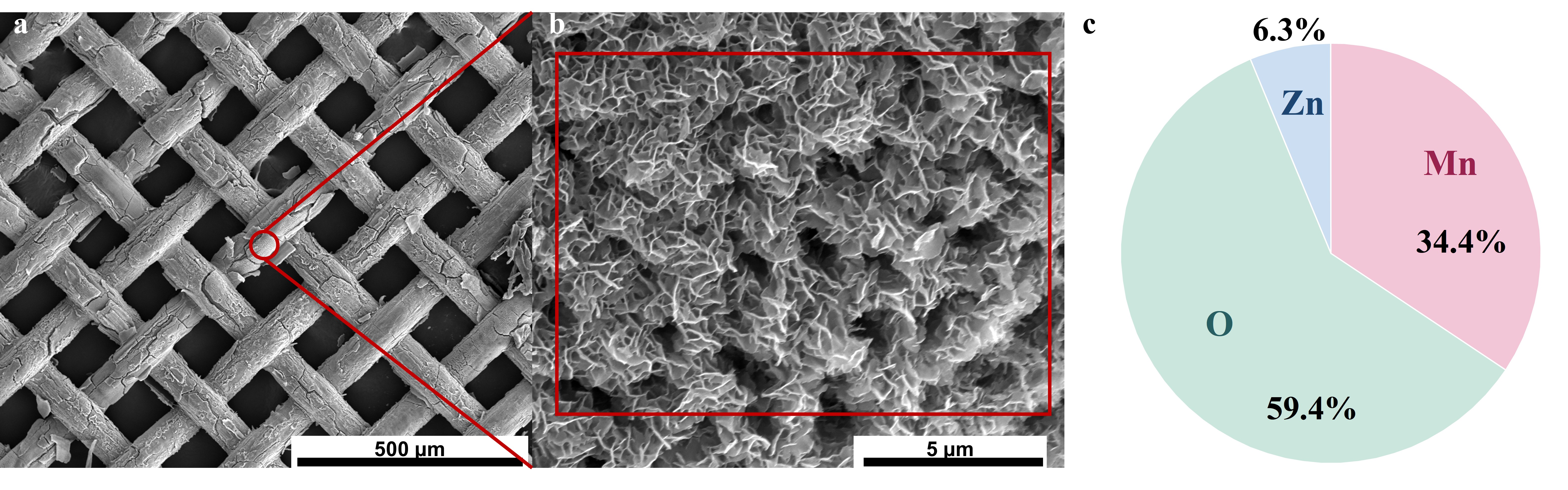


**Fig. S31** SEM images of the SSM cathode from the M20 group after activation, at **a** 250×, and **b** 20000× magnifications. **c** Element composition of selected aera obtained from EDS mapping

**Fig. S32** XRD pattern of the SSM cathode obtained from M20 group after activation


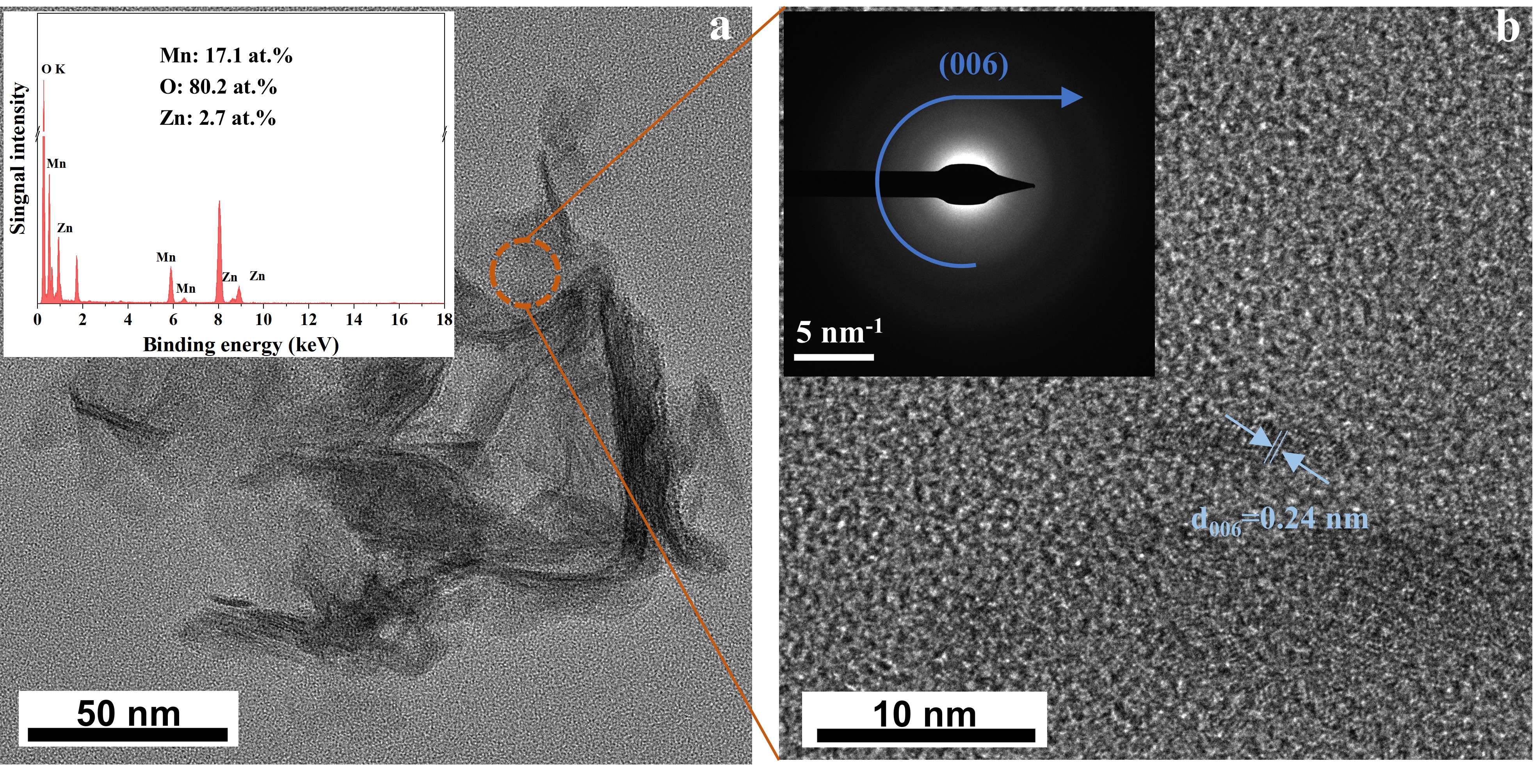


**Fig. S33 a** TEM image of the new phase on the surface of SSM cathode obtained from M20 group after activation, with corresponding EDS point-scan analysis. **b** High-resolution TEM image and the corresponding SEAD pattern


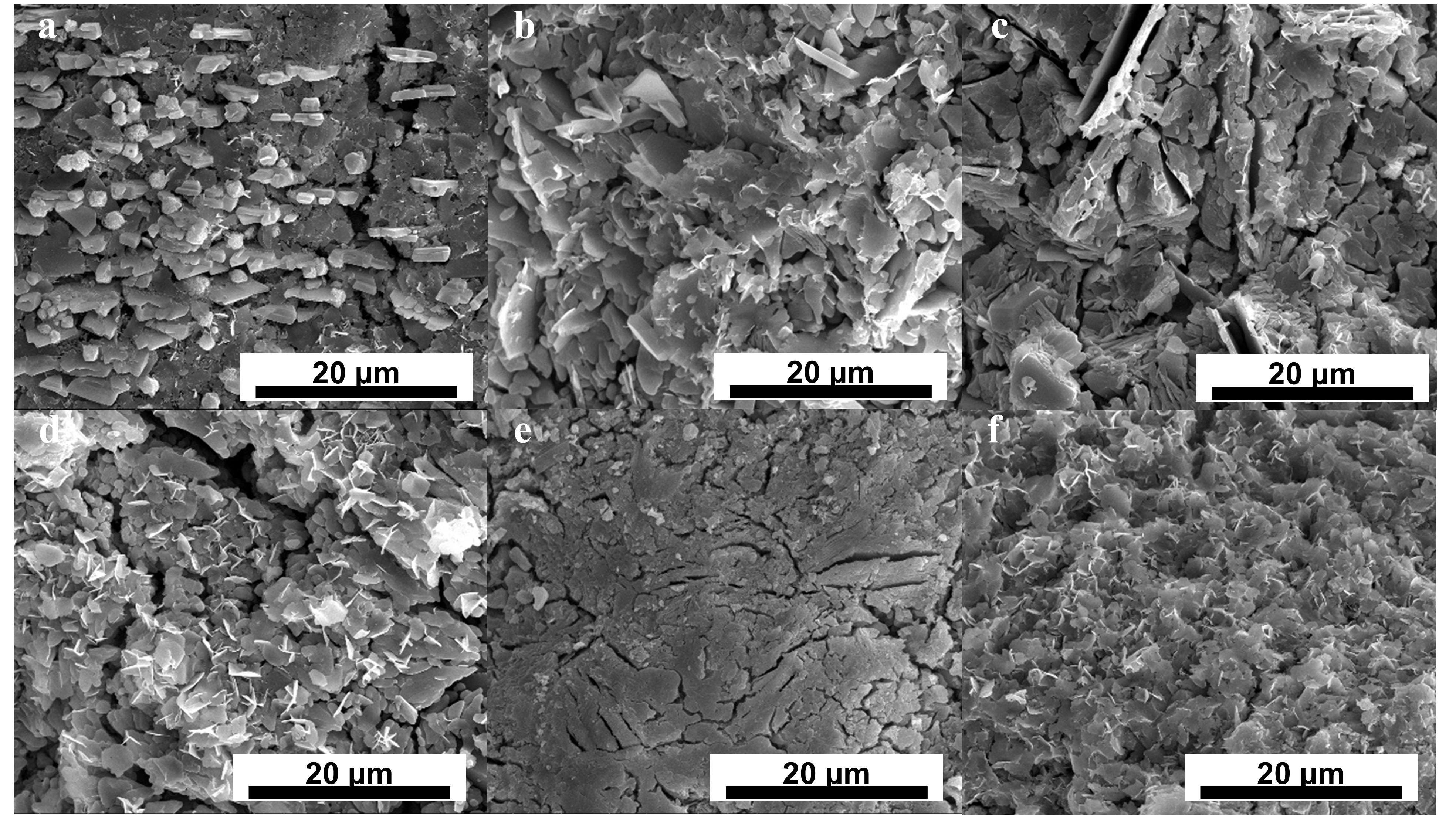


**Fig. S34** SEM images of ACSs from the M20 group at different states: **a** pristine, **b** after the 1^st^ full discharge, **c** after the 2^nd^ full charge, **d** after the 2^nd^ full discharge, **e** after the 5^th^ full charge, **f** after the 5^th^ full discharge


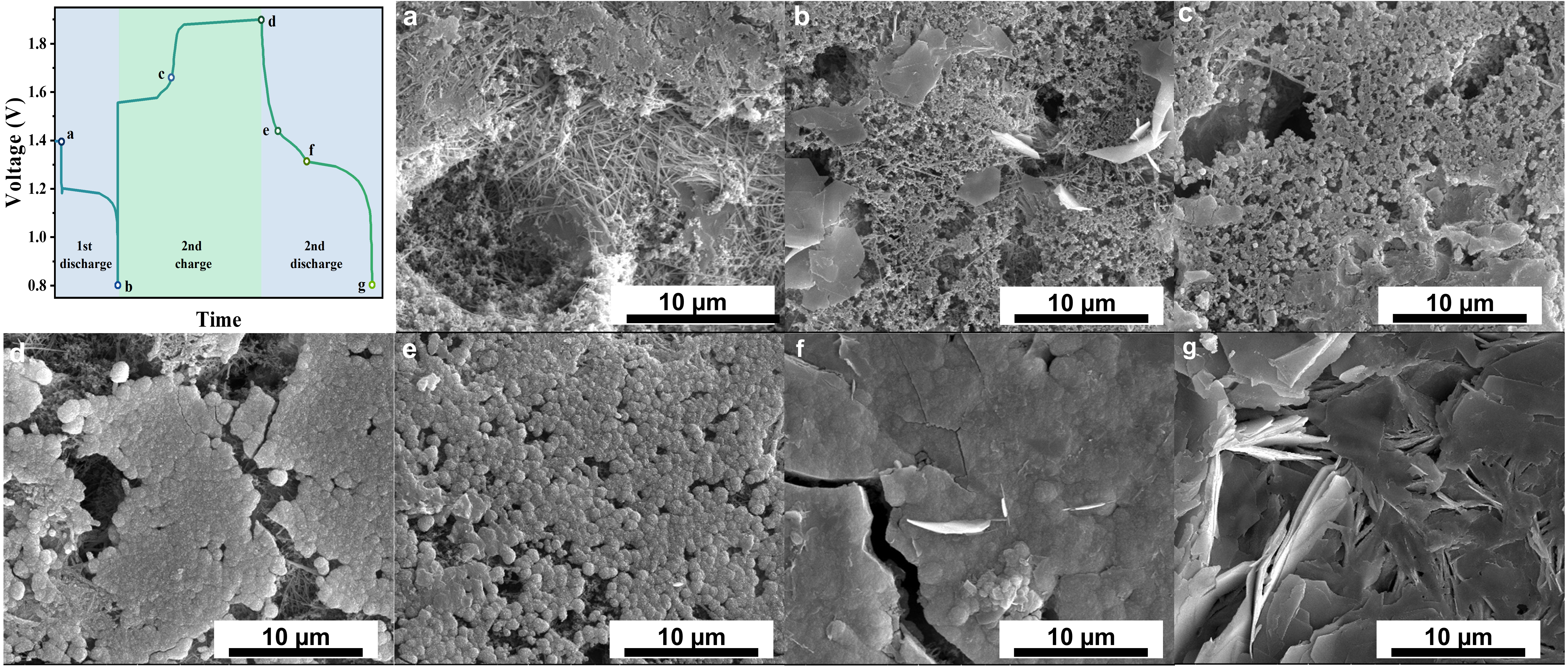


**Fig. S35** SEM images of cathodes from the M20 group at different stages **a**-**g** during the charge-discharge process, as indicated in the corresponding schematic

**Fig. S36** XRD patterns of cathodes from the M20 group at (**a)-(g**) stages

**Fig. S37** LSV curves of CS and ACS group at a scan rate of 0.2 mV s^-1^

**Fig. S38** XRD patterns of cathodes from the M20 group at different activation cycles

**Fig. S39** XRD patterns of the SSM cathode from the M20 group after activation at stages Ⅰ-Ⅵ

**Fig. S40** Specific capacity evolution during capacity-gaining process for groups using CS, ACS, and CS with ACS placed away from the electrodes in the electrolyte

# **S3 Supplementary discussion**

## Note on the Charge/Discharge Mechanism in Fig. 5b, c

To further elucidate the specific charge/discharge mechanism of the birnessite-MnO_2_, XRD patterns of the SSM cathode (without α-MnO_2_) from the M20 group after activation were collected at stages Ⅰ-Ⅵ (same state in Fig. 5b**)**. In this case, the precipitation of birnessite-MnO_2_ still occurs, allowing its role to be specifically examined. Similar to the scenario where the cathode contains α-MnO_2_, the formation of ZnMn_2_O_4_ confirms the intercalation/deintercalation of Zn^2+^ during the discharge/charge process when only birnessite-MnO_2_ is present (Fig. S39). Moreover, the process is accompanied by the deposition and dissolution of ZSH, indicating that H^+^-involved reactions also take place in the cathode containing only birnessite-MnO_2_.
